# Adapting Psychological Therapies for Individuals With Intellectual Disabilities: A Systematic Review

**DOI:** 10.1002/cpp.70202

**Published:** 2026-01-07

**Authors:** Andrew C. Poku, Kylie M. Gray, Olivia Hewitt, Peter E. Langdon

**Affiliations:** ^1^ Centre for Research in Intellectual and Developmental Disabilities The University of Warwick Coventry UK; ^2^ Intellectual Disabilities Research Institute (IDRIS) The University of Birmingham Birmingham UK; ^3^ Department of Psychiatry, School of Clinical Health Sciences at Monash Health Monash University Clayton Victoria Australia; ^4^ Berkshire Healthcare NHS Foundation Trust Learning Disabilities Service, Erlegh House Reading Berkshire UK; ^5^ Herefordshire and Worcestershire Health and Care NHS Foundation Trust Worcester UK; ^6^ Birmingham Community Healthcare NHS Foundation Trust Birmingham UK

**Keywords:** adaptation, intellectual disabilities, mental health, modification, psychological therapy, psychotherapy

## Abstract

The aim of this systematic review was to identify how psychotherapies have been adapted to suit the needs of people with intellectual disabilities. A comprehensive literature search garnered 39,777 studies, which were screened for eligibility. Systematic searches, following PRISMA guidelines, identified 122 studies that met eligibility criteria, and findings were synthesised using framework synthesis. Six overarching adaptation categories were identified: (1) multisensory methods, (2) activities, (3) communication, (4) delivery medium, (5) additional support and (6) structure. Adaptations were primarily used to increase engagement, promote retention of information and improve communication between clients and clinicians. While a group of adaptations that aimed to improve the acceptability and accessibility of psychotherapy for people with intellectual disabilities were identified, larger studies are required to understand their efficacy.

## Summary

- People with intellectual disabilities have been historically neglected in both research and practice, which hindered the development and use of psychotherapy with this population.
- Psychotherapy must be adapted for people with intellectual disabilities; however, the nature and degree of these adaptations and their effectiveness remains unclear.
- We reviewed the literature and developed a psychotherapy adaptation framework.

## 1 Introduction

### 1.1 Mental Health in Individuals With Intellectual Disabilities

An intellectual disability is characterised by a level of general intellectual functioning and adaptive behaviour that falls two or more standard deviations below the average score on standardised tests (Luteijn et al. 2020; World Health Organisation 2022). There is evidence that the prevalence of mental health problems within this population is 3–4 times greater than the general population (Pouls et al. 2022), leading to questions regarding the aetiology of mental health problems, as well as the effectiveness of mental health treatments. A combination of biopsychosocial factors uniquely experienced by these individuals may contribute to an increased risk of developing a mental health problem relative to the general population (Deb et al. 2001; Luteijn et al. 2020). For example, Lehotkay et al. (2009) indicated that the prevalence of mental health problems may increase with severity of intellectual disability, while an increased probability of experiencing stressful life events is also a likely risk factor (Hulbert‐Williams et al. 2014; Hulbert‐Williams and Hastings 2008). Despite the higher rates of mental health problems within this population, primary and secondary mental health care is insufficient for this group (Patterson and Golightly 2023; Pouls et al. 2022). This is partially due to a lack of treatment adapted to accommodate the needs of this population, hindering the accessibility of mental health services, and driving a lower uptake of mental health services compared to the general population (Patterson and Golightly 2023; Pouls et al. 2022; Whittle et al. 2018).

Psychological treatment has been defined as, ‘an intervention consisting of specific actions between a person or persons and a mental health professional or designee, with the intent of engaging cognitive, emotional, behavioural, or interpersonal processes, in the service of modifying health or functional outcomes, and whose core assumptions about its procedures and mechanisms of change are founded in psychological science and consistent with scientific understanding’ (Tolin et al. 2025, 213). Although this definition anchors psychological treatment within a scientific framework, reinforcing psychology as an evidence‐based discipline, it excludes some forms of treatment important in real‐world practice, such as those using technology (e.g., computer‐derived treatment without a provider, artificial intelligence [AI] and transcranial magnetic stimulation), and arguably, non‐specific counselling due to ambiguity around what constitutes ‘specific actions’ (McQuaid 2025). Nevertheless, this definition provides a useful foundation for evaluating empirically supported treatments.

Historically, people with intellectual disabilities were viewed with *therapeutic disdain*; they were considered unable to benefit from psychological therapy due to the nature of their disability (Beail 1998; Bender 1993; Ee et al. 2022; Jamieson and Mason 2019; Tapp et al. 2023). Furthermore, mental health treatments for this group often relied on psychotropic medications rather than psychotherapy (Deb et al. 2023; Sheehan et al. 2015). This hindered the development and use of psychotherapy with this population, compounding uncertainties about the most appropriate therapeutic models (Syed et al. 2020). However, and more recently, there is evidence that psychotherapies are likely helpful for individuals with intellectual disabilities, signifying the increasing importance of offering psychotherapy or nondrug‐based interventions for this group, but substantial uncertainty remains due to the lack of well‐designed and appropriately powered clinical trials (Baxter and Cain 2006; Cottis 2009; Tapp et al. 2023). Nevertheless, psychotherapies need adapting to meet the needs of this population (Dagnan et al. 2023; Surley and Dagnan 2019), even though frameworks as to how to do this have been proposed quite some time ago (Hurley et al. 1998). However, it is the case that there remains a lack of accepted methods for making evidence‐based adaptations.

### 1.2 Adapting Psychotherapy to the Needs of Individuals With Intellectual Disabilities

Hurley et al. (1998) published a framework of therapy adaptations over 25 years ago, Table 1. Since then, several studies have described or tested adaptations to therapy for individuals with intellectual disabilities, for example, Hagiliassis et al. (2005) and Dagnan et al. (2023), although mainly focused upon cognitive behavioural therapy (CBT; Dagnan et al. 2023; Bruce et al. 2010; Vereenooghe et al. 2016; Vereenooghe et al. 2015). Nevertheless, the majority of adaptations to psychotherapy for people with intellectual disabilities are based on clinical experience and opinion rather than drawn from experimental studies evidencing effectiveness (Dagnan et al. 2023).

**TABLE 1:** Summary of adaptations to psychotherapies (Hurley et al. 1998).

Adaptation	Definition/example
Simplification	Reduced usual technique in complexity; break down intervention into smaller chunks, shorter length of sessions
Language	Reduce level of vocabulary, sentence structure and length of thought. Use short sentences; use simple words
Activities	Augment typical techniques with activities to deepen change and learning. Add drawings, homework assignments
Developmental level	Integrate developmental level into presentation of techniques and material. Use games; assess development into relevant social issues
Directive methods	Due to cognitive limitations, must be more direct. Outline treatment goals, progress, give extra ‘visual’ guides
Flexible methods	Adjust usual techniques to suit cognitive level and lack of progress. Draw from other modalities
Involve caregivers	Use family, support staff to help with change. Assign homework or rehearsals at home with the help of staff or family
Transference/countertransference	Attachments are stronger, quicker; therapist reactions similar to parental view. Therapists urged to be stronger in boundaries and to ensure peer supervision
Disability/rehabilitation approaches	Issue of disability must be addressed with treatment. Therapists must raise issues and support positive self‐view

Attempts have been made previously to synthesise the literature about how to adapt psychotherapies for individuals with intellectual disabilities. In their systematic review, Surley and Dagnan (2019) reviewed 23 studies published from 2005 to 2017 about CBT across various clinical contexts. They categorised adaptations according to Hurley et al.'s (1998) framework and reported that activities—methods/techniques that help enhance learning and apply new skills in clients—were incorporated in 21 (91%), simplification of processes in 16 (70%), involvement of carers in 16 (70%), adaptation to developmental level in 15 (65%), adaptation of language in 11 (47%), flexibility in 10 (43%) and use of directive methods in 7 (30%) of the included studies. Two categories, consideration of transference/countertransference and consideration of disability/rehabilitation approaches, were not addressed. Surley and Dagnan (2019) concluded that a more systematic approach regarding the reporting of adaptations is necessary, emphasising that their findings do not constitute a comprehensive review of beneficial adaptations.

Expanding upon the work of Surley and Dagnan (2019), the aim of this systematic review was to synthesise how all types of psychotherapies, not only CBT, were adapted within studies involving individuals with intellectual disabilities in order to address the following research question, ‘in what ways have psychological therapies been adapted to suit the needs of people with intellectual disabilities?’ The findings from eligible studies were synthesised using framework synthesis to develop a framework with higher replicability; an initial framework was created based upon the National Institute for Health and Care Excellence (2016) guideline for adapting psychotherapy for people with intellectual disabilities.

## 2 Method

Our systematic review was conducted in accordance with the Preferred Reporting Items for Systematic Reviews and Meta‐Analyses (PRISMA) guidelines 2020. The protocol was prospectively registered (PROSPERO Registration Number: CRD42023476883).

### 2.1 Study Eligibility Criteria

Studies were included if all the inclusion criteria were met and none of the exclusion criteria were met.

#### 2.1.1 Inclusion Criteria

Studies were included if (1) study participants had an intellectual disability, evidenced by data to indicate that participants had an IQ < 70, or mention of inclusion of participants with an intellectual disability, (2) participants were older than 5 years of age, (3) at least 50% of the sample had an intellectual disability, (4) studies included information about adaptations to psychotherapies for mental health in individuals with intellectual disabilities and (5) experimental, quasi‐experimental studies, qualitative studies and clinical case studies with relevant mental well‐being outcomes, or articles, including the grey literature (e.g., unpublished theses), with descriptive information regarding adaptations to therapy even without outcome data.

#### 2.1.2 Exclusion Criteria

Studies were excluded if (1) studies were solely concerned with behavioural interventions that were not used for mental health (e.g., applied behavioural analysis or behaviour modification for skills teaching), (2) studies were about specific learning difficulties such as specific reading and writing disorders and (3) studies only included pharmacological or other medical treatments for mental health.

### 2.2 Information Sources

#### 2.2.1 Databases

PsycINFO, MEDLINE and CINAHL were searched through OVID. PsycINFO was searched on 31 August 2023, MEDLINE was searched on 25 August 2023 and CINAHL was searched on 30 August 2023. An updated search using the same databases commenced on 9 May 2024, 14 Oct 2024 and 7 May 2025.

#### 2.2.2 Search Strategy and Search Term

Search terms used for all databases related to intellectual disabilities, psychotherapy and mental health. The research team collaborated on the production of the search strategy, favouring an approach that broadened the scope of the literature to be screened and ensure that no manuscripts were missed. There were no restrictions placed upon the searches. Search terms were comprised of synonyms related to intellectual disability, psychological intervention and mental health. The full list of search terms can be found in the Supporting Information.

#### 2.2.3 Selection Process

**FIGURE 1:**
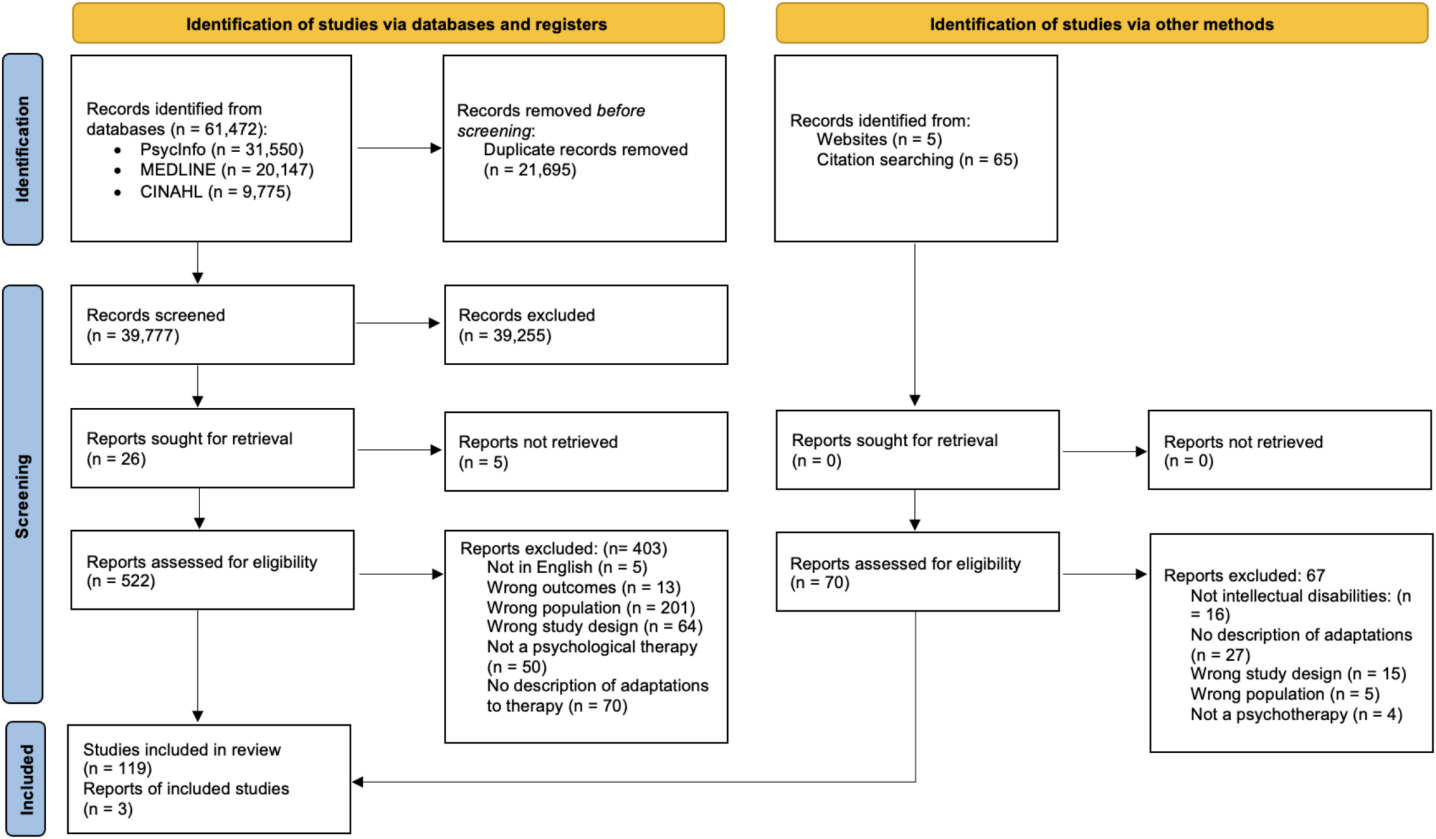
PRISMA flow chart.

Initially, the results from all database searches were exported to Covidence (Covidence. 2023). This is a software used for collaborative systematic reviews. There was a total of 56,976 papers identified, and 19,337 duplicates identified using Covidence, which left a total of 37,639 studies to be screened. A total of 12.35% (*n* = 4647) of results were independently reviewed by OH (co‐reviewer) at the title and abstract stage of screening. A total of 37,126 papers were excluded after title and abstract screening, leaving 513 to be screened at the full‐text stage. The screening process was blinded and was conducted using Covidence. Agreement at the title and abstract stage was 93% (Cohen's *k* = 0.46), any conflicts were discussed until full agreement was reached at 100% (Cohen's *k* = 1; *n* = 4647). Full‐text screening was conducted for all papers by AP, with 28.27% (*n* = 145) of papers screened by OH independently. Agreement after the full‐text stage was initially 73% (Cohen's *k* = 0.46), with all conflicts resolved after discussion leading to 100% agreement (Cohen's *k* = 1; *n* = 145). An updated screening of the same databases, with a restricted search of articles published from 1 February 2024 to 9 May 2024, 1 April 2024 to 14 October 2024 and 1 November 2024 to 7 May 2025, garnered a total of 4496 papers and 2358 duplicates, which were screened using Rayyan (Ouzzani et al. 2016). A total of 2138 articles were subsequently screened, leading to an exclusion of 2129 papers, leaving nine papers eligible for inclusion. After full‐text screening was completed, a total of 122 studies were included within the present systematic review. One study (Langdon et al. 2024a; Langdon et al. 2024b) was published in a peer review journal and as a peer reviewed report by the funder and was counted twice in the total number of included studies. A PRISMA flow diagram can be found within Figure 1.

### 2.3 Data Extraction

Data extraction was conducted by AP, with 10.66% (*n* = 13) of papers randomly selected for review by an independent second reviewer (OH). Any disagreements were discussed until full agreement was reached at 100% (*k* = 1). The data extraction table was based on the Cochrane template for RCTs (Higgins et al. 2024), with appropriate adaptations made for use with both qualitative and quantitative studies. The data extraction table can be seen in Table S1. Data items were as follows:

#### 2.3.1 Study Type

Relevant study characteristics, where applicable, included the type of publication (e.g., book and article), the number of and detailed description of the study arms (e.g., experimental and control groups). Additionally, the research methodology employed (e.g., quantitative, qualitative or mixed‐methods approaches) was documented. Nonempirical articles and grey literature, such as unpublished theses or reports, were considered when relevant to the review's objectives.

#### 2.3.2 Participants

Relevant sample characteristics included the number of participants, age (mean/standard deviation/range), severity of intellectual disability (Full‐Scale IQ if provided) and mental illness/mental health condition included within the sample.

#### 2.3.3 Intervention

Intervention information included the type/name of the intervention, a brief description of what the intervention entailed, session length, intervention length and setting (if provided).

#### 2.3.4 Adaptation

Summary of adaptations to psychotherapy mentioned within the text. This included mode of delivery and descriptions of adaptations (e.g., pictures, simplified language and inclusion of carers).

#### 2.3.5 Future Adaptation Recommendations

Detail of recommendations of adaptations for future interventions.

#### 2.3.6 Outcome

Changes in mental health symptomatology (if applicable).

#### 2.3.7 Follow‐Up

Information about follow‐up duration and intervention effects at follow‐up.

#### 2.3.8 Quality

The results from the Mixed‐Methods Appraisal Tool (MMAT; Hong et al. 2018) and JBI Checklist for Case Reports (Munn et al. 2020).

### 2.4 Quality Appraisal

The present systematic review included qualitative, quantitative and mixed‐method study designs. The quality and bias of these studies were assessed using the MMAT (Hong et al. 2018). This tool was thought to be most appropriate due to the range of study types that were included in the systematic review. This used a 6‐point scale from 0 to 5. For descriptive case studies, the JBI Checklist for Case Reports (Barker et al. 2020) was used. This used a 9‐point scale from 0 to 8. Overall, 87 studies were assessed using the MMAT, and 14 were assessed using JBI for case series. Quality of selected studies was assessed independently by OH and AP, with OH assessing 13 (10.66%) of articles. Any disagreements were discussed until full agreement was reached at 100% (*k* = 1).

### 2.5 Data Synthesis

Data were synthesised across the studies using framework synthesis following a five‐step procedure (Brunton et al. 2020): (1) familiarisation of the literature, (2) this was then organised into a framework initially based upon the National Institute for Health and Care Excellence (2016) recommendations for adapting therapy for people with intellectual disabilities, (3) indexing, (4) charting by using descriptive content analysis to identify and count categories of types of adaptations to psychotherapy for people with intellectual disabilities and (5) mapping and interpretation as well as revision of the framework. This method was chosen to enhance the replicability of the framework and increase the validity of results obtained.

#### 2.5.1 Familiarisation

National Institute for Health and Care Excellence (2016) guideline pertaining to conducting psychotherapy with people with intellectual disabilities was reviewed and used to develop an initial framework of adaptations. This guideline recommended that a mental health assessment should inform the psychological intervention and any adaptations. Adaptations should (1) be tailored to preferences, level of understanding and strengths and needs, (2) consider physical, neurological, cognitive or sensory impairments and communication needs, (3) consider the need for privacy and (4) use an individual's intervention delivery preferences (e.g., face‐to‐face or remotely).

The guideline recommended that clinicians should collaborate with clients and their family members, carers or care workers to develop and agree (1) the intervention goals, (2) an understanding of how the person expresses or describes emotions or distressing experiences, (3) the structure, frequency, duration and content of the intervention, including its timing, mode of delivery and pace, (4) the level of flexibility needed to effectively deliver the intervention and (5) how progress will be measured and how data will be collected (e.g., visual representations of distress or well‐being).

Included within the guideline was a recognition that people with intellectual disabilities may need a more structured support to practise and apply new skills in everyday life between sessions. Therefore, in collaboration with clients, clinicians may (1) provide additional support during meetings and plan activities between meetings and (2) ask a family member, paid or unpaid carer, to provide support and assistance (e.g., reminders) to practise new skills between meetings.

The guideline was used to develop the initial framework (Table S2) as defined below and included (1) targeted mental health problem, which was initially included as a category, reflecting the emphasis placed in existing guidance, by the National Institute for Health and Care Excellence (2016), on using mental illness diagnoses to inform adaptations made to psychotherapies, (2) contextual alterations (i.e., changes in therapy setting) or adaptations required to accommodate for the personal preferences of clients, for example, including extra introductory sessions to build rapport with clients (Digman 2021), (3) language: the simplification of language, (4) support: inclusion of supporters during therapy, whether family members or other caregivers, (5) mode of delivery: the modality of an intervention defined as (1) in person, (2) online or (3) telephone and (6) intervention flexibility: alterations to the structure of therapy such as the number of sessions or the addition of more breaks.

#### 2.5.2 Indexing and Charting

Table S2 provided a foundation for identifying adaptations within the literature. The framework provided predefined categories and criteria, which were applied during the coding process to classify and organise types of adaptations observed in the included studies. Content analysis was employed to ensure a systematic approach, with each identified adaptation categorised under the relevant headings of the initial framework. The reported adaptations were grouped into six overarching categories: (1) multisensory methods, (2) activities, (3) communication, (4) delivery medium, (5) additional support and (6) structure. Each overarching category comprised of associated subcategories, which were the specific adaptations reported within the included studies. Adaptations were categorised and counted under the relevant subcategory. Percentages of subcategories were calculated relative to the total number of adaptations within their respective overarching category.

As stated earlier, the National Institute for Health and Care Excellence (2016) guideline suggested that adaptations should be informed by the targeted mental health problem; thus, this was inputted as a category within the initial framework, with the assumption that adaptations would vary depending on the targeted mental health problem.

For adaptations that could fall under multiple categories, relevant modifications were made to the framework for enhanced clarity. For example, as noted in the review by Surley and Dagnan (2019), changes to session length could have been categorised as either simplification or flexible methods. In these cases, importance was placed on clearly defined categories, with clear distinctions between them; when necessary, categories were expanded to encompass a greater number of identified, and associated, adaptations.

#### 2.5.3 Mapping and Interpretation

During the data synthesis stage, categories were developed from the characteristics of the included studies. These categories were examined in reference to National Institute for Health and Care Excellence (2016) guidance, as well as the included studies, with an aim of providing a detailed description of the nature and frequency of the adaptations. Table S3 shows the final framework for adaptations.

## 3 Results

A total of 522 studies were eligible for full‐text screening, of which 122 were included within the review, Figure 1. Many studies were excluded because at least 50% of their participant sample did not have an intellectual disability and instead had autism or a specific learning difficulty (e.g., dyslexia). Other reasons for exclusion included a lack of information about adaptations to psychotherapies for mental health, a focus upon applied behaviour analysis that was not being used to treat mental health problems or the study was published in a language other than English. All of the included studies and the associated findings are detailed in Table S1. Those included in our synthesis, and found in Table S1, but not cited below are: Acton et al. (2023); Alilou and Maleki 2024; Ashworth et al. 2017; Baldwin et al. 2021; Barnett 2024; Barrowcliff and Evans 2015; Blakeley‐Smith et al. 2021; Boulton et al. 2018; Browne and Smith 2016; Buhler 2014; Buijs et al. 2021; Campbell and Scarpa 2019; Carrigan and Allez 2017; Carter 2022; Cooney et al. 2017, 2018; Cooper and Frearson 2017; Didden et al. 2019; Dillon et al. 2018; El‐Tahir and Bayley 2017; Essau and Longhi 2013; Evans and Allez 2018; Fernandez et al. 2005; Goad and Parker 2021; Gray et al. 2024; Gregson and Delaney 2021; Haddock et al. 2004; Hartley et al. 2015; Hackett 2012; Hassiotis et al. 2011; Hewitt et al. 2019, 2023; Hoogstad and Mevissen 2024; Hronis et al. 2024, 2019; Jahoda et al. 2024, 2015; Kirk et al. 2014; Lake and MacHale 2022; Lew et al. 2006; Lewis and Rose 2018; Lindsay et al. 2015; Mevissen et al. 2011, 2012, 2020; Montanaro et al. 2024; Moore et al. 1997; Oathamshaw et al. 2013; O'Farrell et al. 2024; Pineda et al. 2023; Porter 2022; Power et al. 2022; Roberts and Kwan 2018; Rossiter et al. 1998; Searle and Borseti 2021; Singh et al. 2021; Stenfert‐Kroese et al. 2016; Ugwu et al. 2022; Unwin et al. 2023; Verberg et al. 2018; Vereenooghe and Westermann 2019; Weber and Streicher 2021; Willner et al. 2011, 2002

### 3.1 Participants

Reports of participant information varied across the 122 included studies. Thirteen studies, such as van Wingerden et al. (2021) and Ashworth et al. (2020), included Full‐Scale IQ scores, while others, such as Dilly (2014) and Ali et al. (2023), relied on case notes and prior diagnoses of intellectual disability. Most of the included studies reported three degree of intellectual disability (e.g., mild and moderate) without IQ scores (*n* = 64), and 22 studies did not specify severity. Nevertheless, five studies included participants with borderline, 66 studies included participants with mild, 56 included moderate and 16 with severe‐profound intellectual disability. The ages of participants ranged from 2 to 72 years, with 14 studies having included participants younger than 18 years.

### 3.2 Methods

The methodologies of included studies were varied; the majority were single‐arm within‐subjects pre–post designs (*n* = 26) and case study designs (*n* = 38). Case study designs included descriptive case studies (*n* = 15), quantitative case studies (*n* = 9), case studies using mixed methods (*n* = 4), pre–post case studies (*n* = 7) and multiple baseline case study designs (*n* = 3). Other studies were randomised controlled trials (RCT, *n* = 14), nonrandomised between‐group designs with two groups (*n* = 4) or three groups (*n* = 3), cohort studies (*n* = 3), one longitudinal study, RCT protocols (*n* = 5), feasibility studies (*n* = 11), one presentation, one descriptive report, one randomised controlled quasi‐experimental design, one manual and one exemplar. Most researchers opted to use quantitative methods (*n* = 44), whereas there were 33 qualitative and 26 mixed‐methods studies. The number of recruited participants rarely exceeded 25, although some studies, such as Willner et al. (2013), Ho et al. (2020) and Jahoda et al. (2017) were able to recruit greater numbers from multiple centres (*N* = 212, *N* = 109 and *N* = 141, respectively). The number of participants included within RCTs ranged from *n* = 14 to 212, with the median being *n* = 29, while RCT protocols had a target median sample size of 167 participants, indicating a desire for studies with larger sample sizes to be conducted.

### 3.3 Quality

#### 3.3.1 MMAT

Studies assessed using the MMAT revealed quality ratings within the moderate–high range, with the lowest score being 3 (*n* = 17). This meant that, overall, studies had clear research questions, participants that were representative of the target population, and methodologies that were appropriate to answer the research questions. Key issues observed within lower quality studies included (1) incomplete outcome data, for example, Wilson et al. (2020) and Verhagen et al. (2023), who had missing data from participants; (2) samples that may not be representative of the target population, for example, Ashworth et al. (2021), who only included male participants; and (3) unclear integration of qualitative and quantitative results, such as Jones et al. (2021).

#### 3.3.2 Joanna Briggs Institute (JBI) Case Series

Descriptive case studies assessed using JBI case series had scores that ranged from 5 to 8, indicating a moderate–high level of quality for included studies. This meant that, overall, studies included adequate clinical information and clearly reported outcomes. One study, Abrego (2020), received a score of 2, which was due to an unclear description of participant demographic characteristics, history, current clinical condition and unclear assessment methods, description of treatment procedures and adverse/unanticipated events.

### 3.4 Interventions

As stated earlier, the National Institute for Health and Care Excellence (2016) guideline suggested that adaptations should be informed by the mental health problem; thus, this was inputted as a category within the initial framework, with the assumption that adaptations would vary depending on the targeted mental health problem. However, during the data synthesis stage, and unsurprisingly, it was apparent that adaptations were predominantly made to address the severity of intellectual disability, rather than the mental health problem that an intervention aimed to treat. For example, Florez and Bethay (2017) adapted handouts and recordings to contain more concrete images and simpler language to match their participant's intellectual functioning. Furthermore, Dilly (2014) used images of scenery, selected by their participant, in conjunction with a scented fir cone for additional stimulation, to simulate a ‘safe‐space’ environment and support the participant in overcoming difficulties with visualisation. Mental health problems did, however, influence the chosen psychotherapy modality, and for this reason, targeted mental health problem was removed as a category in our framework. Instead, types of psychotherapies were categorised, counted and grouped by mental health problem, and percentages were subsequently calculated with reference to the targeted mental health problem. This was done to gain an understanding of the preferred treatment modalities for mental health problems experienced by people with intellectual disabilities.

Intervention types were identified and counted, alongside which type of mental health problem they were used to treat. For studies about anxiety/stress, the majority used CBT (50.00%), with one intervention that combined CBT and mindfulness. Other interventions included mindfulness (13.04%), dialectical behaviour therapy (DBT; 8.69%), mentoring (4.35%), art therapy (2.17%), compassion‐focussed therapy (CFT; 2.17%), robot‐mediated therapy (2.17%), sandtray therapy (2.17%), an unspecified videoconference‐mediated therapy (2.17%), befriending/peer support (2.17%), narrative therapy (4.35%), behavioural activation (2.17%) and acceptance and commitment therapy (2.17%). Mentoring and befriending/peer support were categorised separately due to differences in the nature of relationships and intervention goals. Research indicated that befriending exists on a spectrum, ranging from reciprocal, equal relationships resembling natural friendships to more professional, therapeutic relationships with an emphasis on goal attainment, more closely resembling mentoring (Ali et al. 2020; Thompson et al. 2016).

For studies about anger, the majority made use of CBT (61.54%), followed by mindfulness (15.38%), DBT (7.69%), strength‐based therapy (7.69%) and an unspecified videoconference‐mediated therapy (7.69%). For studies about depression/low mood, the majority used CBT (25.71%), with one that used a mindfulness‐based CBT. Other interventions for depression/low mood included DBT (11.43%), mindfulness (8.57%), art therapy (8.57%), compassion‐focused therapy (8.57%), mentoring (5.71%), behavioural activation (11.43%), one used an unspecified videoconference‐mediated therapy, one used rational emotive behaviour therapy, one used a multidisciplinary approach combining psychological (unspecified therapy), pharmacological and behavioural interventions, one used acceptance and commitment therapy and one used online mental health interventions called *Moodgym*, an online self‐management programme based on principles from CBT and interpersonal therapy and *iFightDepression*, an online programme designed to support patients with intellectual disabilities who were enrolled in psychotherapy (Vereenooghe et al. 2021).

For studies about trauma/post‐traumatic stress disorder (PTSD), the majority used eye movement desensitisation and reprocessing (EMDR; 56.52%), followed by CBT (13.04%), narrative therapy (8.70%), one used DBT, one used sandtray therapy, one combined systemic therapy, narrative therapy and interpersonal therapy, one used systemic team formulation‐trauma informed care and one used progressive counting. For psychosis/schizophrenia, interventions included CBT (20.00%), drama therapy (20.00%), sandtray therapy (20.00%), befriending/peer support (20.00%) and acceptance and commitment therapy (20.00%). For studies about emotional dysregulation, most studies used DBT (33.33%), followed by CBT (16.67%), mindfulness (16.67%), strength‐based therapy (16.67%) and narrative therapy (16.67%). For personality disorders, the majority used DBT (66.67%), followed by mindfulness (33.33%). For confidence/low self‐esteem, five used CBT.

The National Institute for Health and Care Excellence (2016) recommended the use of adapted CBT for depression, relaxation therapy for anxiety and graded exposure techniques for anxiety or phobia. Although CBT was a commonly chosen treatment modality for individuals with depression within the included studies, it was also used for other mental health problems, such as anxiety, trauma and anger, emphasising the versatility of the approach. Relaxation techniques were incorporated into a variety of psychotherapies, such as EMDR (Porter 2022), CBT (Panditaratne et al. 2022) and mindfulness (Yildiran and Holt 2015), and were important tools for regulating emotion and reducing anxiety. Graded exposure techniques were also used in CBT interventions for people with anxiety, although these were less common.

### 3.5 Adaptations to Psychotherapy

As stated earlier, the initial framework developed using the National Institute for Health and Care Excellence (2016) guidance (Table S2) was used to identify and categorise adaptations that were present within the literature. However, the initial framework did not sufficiently capture the breadth of methods used by researchers to adapt interventions to meet individual needs, categorise types of supporters or document the various structural changes to therapy reported in the literature. Additionally, although the National Institute for Health and Care Excellence (2016) guidance broadly emphasised the importance of considering the cognitive and emotional challenges that people with intellectual disabilities may experience, limited detail about the specific adaptations required to address these challenges was included. This led to uncertainties when coding, and as a result, the framework was modified to reflect the diversity of adaptations documented within the literature. For instance, some adaptations fell into multiple categories, which occurred because of unclear distinctions between categories, in these cases, relevant modifications were made to the framework for enhanced clarity. For example, as noted in the review by Surley and Dagnan (2019), changes to session length could have been categorised as either simplification or flexible methods. This is because Surley and Dagnan (2019) partially defined simplification as the shortened length of sessions, and flexible methods as the adjustment of usual techniques. However, our review found that session lengths were not always reduced, and session length modifications did not always reflect a flexible approach to therapy, making these categorisations insufficient. Categories were therefore expanded and became more detailed, for example, the overarching category of *structure* was used to denote any adaptations made to psychotherapy to suit the individual needs of clients with intellectual disabilities. This encompassed an array of adaptations such as longer sessions, shorter sessions, more sessions, less sessions, slower pace and inclusion of breaks. This provided clearer distinctions between categories and emphasised the importance of detailed adaptation reports.

The evolution of the initial framework was guided by content analysis. For example, the authors of several studies emphasised the importance of incorporating audio‐visual methods during therapy and explained that this approach was used to enhance engagement and understanding, a concept not explicitly included within the initial framework. This led to the identification of a new category, ‘multi‐sensory methods’, which was added to the framework, and sub‐categories included visual methods, audio methods, olfaction and other (e.g., somatosensory and unspecified). These categories were then used to synthesise the findings, and subcategories/codes were used to score and count the referenced adaptations. Any adaptations not included in the initial codes were added to the framework and applied to all included studies. The final coding tool can be found in Table 2.

**TABLE 2:** Final coding tool.

Category	Subcategory
**Multisensory methods**	Visual
	Audio
	Olfaction
	Other (e.g., somatosensory and unspecified)
**Activities**	Role play
	Art
	Games
	Other physical activities (e.g., exercise and sand tray)
**Communication**	Simplified language
	Repetition
	Concrete examples
	Non‐verbal components
	Prompts
	Positive reinforcement
	Directive approach
**Delivery medium**	Face‐to‐face
	Online
	Video call/telephone
	Multimodal
	Technology
	Multidisciplinary approach
	Individual setting
	Group setting
	Flexible environment
**Additional support**	Paid carers
	Relatives/significant other
	Peer support
**Structure**	
	Shorter sessions
	Longer sessions
	More sessions
	Less sessions
	Increased frequency
	Decreased frequency
	Slower pace
	Content reduction
	Breaks
	Structured approach
	Flexible approach
	Additional psychoeducation
	Frequent progress tracking
	More homework
	Individual needs
	Reduced group size
	Additional resources
	Relaxation exercises

Forty‐five subcategories were identified, categorised and scored within the 122 included studies. These adaptations were grouped into six overarching categories, and percentages of subcategories were calculated relative to the total number of adaptations within their respective overarching category.

A summary of the frequency and nature of adaptations can be found in Table 3. Our findings using our revised framework are described below.

**TABLE 3:** Frequency and nature of adaptations.

Category	Subcategory	Adaptation frequency count^a^
Multisensory methods	Visual	81 (68.64%)
	Audio	23 (19.49%)
	Olfaction	4 (3.39%)
	Other (e.g., somatosensory and unspecified)	10 (8.47%)
	Total	118 (100%)
Activities	Role play	20 (29.41%)
	Art	8 (11.76%)
	Games	10 (14.70%)
	Other physical activities (e.g., exercise and sand tray)	30 (44.12%)
	Total	68 (100%)
Communication	Simplified language	68 (45.03%)
	Repetition	38 (25.17%)
	Concrete examples	21 (13.91%)
	Non‐verbal components	8 (5.30%)
	Prompts	10 (6.62%)
	Positive reinforcement	3 (1.99%)
	Directive approach	3 (1.99%)
	Total	151 (100%)
Delivery medium	Face‐to‐face	102 (43.77%)
	Online	9 (3.86%)
	Video call/telephone	2 (0.86%)
	Multimodal	5 (2.15%)
	Technology	4 (1.72%)
	Multidisciplinary approach	7 (3.00%)
	Individual setting	58 (24.90%)
	Group setting	41 (17.60%)
	Flexible environment	5 (2.15%)
	Total	233 (100%)
Additional support	Paid carers	48 (59.26%)
	Relatives/significant other	28 (34.57%)
	Peer support	5 (6.17%)
	Total	81 (100%)
Structure	Shorter sessions	8 (3.64%)
	Longer sessions	5 (2.27%)
	More sessions	13 (5.91%)
	Less sessions	3 (1.36%)
	Increased frequency	9 (4.09%)
	Decreased frequency	3 (1.36%)
	Slower pace	17 (7.73%)
	Content reduction	4 (1.82%)
	Breaks	8 (3.64%)
	Structured approach	24 (10.91%)
	Flexible approach	16 (7.27%)
	Additional psychoeducation	26 (11.82%)
	Frequent progress tracking	10 (4.55%)
	More homework	14 (6.36%)
	Individual needs	18 (8.18%)
	Reduced group size	6 (2.73%)
	Additional resources	24 (10.91%)
	Relaxation exercises	12 (5.45%)
	Total	220 (100%)

^a^ Percentages of subcategories were calculated relative to the total number of adaptations within their respective overarching category.

#### 3.5.1 Multisensory Methods

The use of multisensory methods refers to methods of conveying information by limiting verbal and written communication, which have been shown to be barriers limiting accessibility of psychotherapy for this population (García et al. 2020). A total of 118 (13.55%) of identified adaptations across studies were coded as multisensory methods. These were visual methods (68.64%), audio methods (19.49%), olfaction (3.39%) and other (e.g., somatosensory and unspecified) (8.47%). These were incorporated into therapy in a variety of ways, for example, the use of a scented fir cone to create a ‘safe space’ to manage client arousal and promote the use of imagery (Dilly 2014), or visual methods to help clients express thoughts and emotions (e.g., Datlen and Pandolfi 2020; Gilrane‐McGarry and Taggart 2007). Multisensory methods were also used as prompts to increase client engagement, aid with memory and explanations of concepts (Didden et al. 2022; Giannaki and Hewitt 2021; Keesler et al. 2023; Porter 2022).

#### 3.5.2 Activities

The use of activities was reported 68 (7.81%) times across the included studies, and involved role play (29.41%), art (11.76%), games (14.70%) and other physical activities (44.12%). For example, Komarek (2020) implemented sandplay during therapy, which was used to help clients to express emotions, and Digman (2021) had tea and cake or went for walks with clients to build rapport. Hagiliassis et al. (2005) used role play as an active learning technique to reinforce learned concepts and offer clients a medium to practise techniques. Ho et al. (2020) incorporated rhythm/dance movements, as well as music games to encourage clients to express thoughts and feelings through non‐verbal channels, while Brown and Hooper (2009) went for walks or included activity‐based exercises within sessions.

#### 3.5.3 Communication

Augmenting communication was reported 151 (17.34%) times across the included studies. These included simplified language (45.03%), repetition (25.17%), concrete examples (13.91%) and non‐verbal components (5.30%), such as nodding of the head and changing of facial expressions to convey understanding of a conversation (Hassiotis et al. 2012). Other methods involved prompts (6.62%), positive reinforcement (1.99%), utterances (e.g., compliments) to provide encouragement to clients and reward understanding of learned concepts/skills during therapy and directive approaches (1.99%). Good communication is an essential aspect of psychotherapy, and so, ensuring that the communication needs of clients are met is paramount (Boardman et al. 2014; García et al. 2020). Adapting how clinicians communicate with clients has been shown to be beneficial for people with intellectual disabilities. For example, the use of shortened, direct sentences with concrete examples aids in the comprehension and retention of information (Didden et al. 2022; Giannaki and Hewitt 2021). Furthermore, repetition of concepts discussed within sessions may enhance moderate cognitive or emotional arousal by repeating mindfulness and relaxation exercises (Craven and Shelton 2020; Prangnell and Green 2008). Croom et al. (2021) used a mixture of both verbal and non‐verbal communication, which also aided in client understanding of information.

#### 3.5.4 Delivery Medium

The medium by which therapy can be delivered was included as an adaptation 233 (26.75%) times across the included studies. Subcategories included face‐to‐face (43.77%), online (3.86%), video call/telephone (0.86%) and multimodal (2.15%). Other subcategories included technology (1.72%), multidisciplinary approach (3.00%), individual setting (24.90%), group setting (17.60%) and a flexible environment (2.15%). A flexible environment refers to changes made to the environment by which therapy is conducted, for example, opting to take clients outdoors (Digman 2021). The vast majority of studies conducted therapy face‐to‐face in an individual setting, which could allow for more time and attention to be spent with clients, enabling the therapist to have better focus on the client's needs (Newlands and Benuto 2020). Conducting therapy in a group setting was also found to be beneficial, as this helped foster an environment that allowed clients to engage with peers and partake in exercises with one another. This in turn could lead to increased enjoyment of therapy, as well as increased retention rates (Croom et al. 2021; Datlen and Pandolfi 2020; Jones et al. 2021). Sessions that were held online or over video call/telephone were not found within the literature as frequently as in‐person sessions.

#### 3.5.5 Additional Support

This category referred to the inclusion of supporters that helped facilitate an intervention and was reported only 81 (9.30%) times across the included studies. This involved paid carers (59.26%), relatives/significant others (34.57%) and peer support (6.17%). Several groups indicated that additional support could benefit people with intellectual disabilities. For example, Weeland et al. (2015) proposed to involve carers in homework exercises to help encourage participants to practise skills learned outside of sessions. Whitehead et al. (2021) included ‘key workers’ as central points of support and contact for families of participants receiving treatment from an interdisciplinary team. Carers in this study played a significant role in the treatment planning for young people with learning disabilities and received ongoing guidance from the multidisciplinary team. Ali et al. (2021) included volunteers from the community, known as befrienders, who were matched with a person with an intellectual disability based on shared interests and availability. The purpose was to provide emotional support and encourage participation in community activities. They found potential psychological and social benefits for both befrienders and individuals with intellectual disabilities experiencing symptoms of depression. In some studies, carers were included to facilitate communication and understanding between therapists and individuals with intellectual disabilities (e.g., Giannaki and Hewitt 2021), as well as aid with personalised treatment (Perera et al. 2022).

#### 3.5.6 Structure

Adapting the structure of therapy was reported 220 (25.26%) times across the included studies. There were a variety of ways therapy structure was adapted, leading to 18 subcategories, which were (1) shorter sessions (3.64%), (2) longer sessions (2.27%), (3) more sessions (5.91%), (4) less sessions (1.36%), (5) increased frequency (4.09%), (6) decreased frequency (1.36%), (7) slower pace (7.73%), (8) content reduction (1.82%), (9) breaks (3.64%), (10) structured approach, which refers to a rigid, systematic approach of therapy with minimal variability, ensuring consistency in treatment delivery (10.91%), (11) flexible approach, which refers to an adaptive approach to therapy that allows for variability in treatment delivery (7.27%), (12) additional psychoeducation (11.82%), (13) frequent progress tracking (4.55%), (14) more homework (6.36%), (15) individual needs, which refers to personalised and unspecified adaptations to therapy that are based on client needs (8.18%), (16) reduced group size (2.73%), (17) additional resources, for example, educational materials, such as leaflets, drawings and worksheets (Karatzias et al. 2019; Rawlings et al. 2021) (10.91%) and (18) relaxation exercises (5.45%).

Examples of adaptations to the structure of therapy are evident in studies such as Kneuer (2014), which recommended shorter and more frequent sessions to allow additional time to establish a therapeutic relationship and improve clients' concentration. Rawlings et al. (2021) and Verberg et al. (2022) adopted a flexible approach, tailoring therapy to individual needs, such as giving clients agency over the topics discussed in sessions/assignments, and allowed clients to choose the medium of therapy delivery. In contrast, Ashworth et al. (2020) and Jones and Finch (2020) incorporated a more structured approach to treatment, favouring consistency between sessions. Additional psychoeducation and resources were also found to be helpful in consolidating learned concepts, and helping clients to understand their mental health (Ashworth et al. 2020; Clapton et al. 2018). Often, these psychoeducational resources would involve pictorial and easy‐read materials (e.g., leaflets and drawings) that were more accessible to people with intellectual disabilities (Jones et al. 2021; Rawlings et al. 2021).

## 4 Discussion

We aimed to understand the ways in which psychotherapies for mental health problems have been adapted to suit the needs of people with intellectual disabilities. At present, there is a lack of generally accepted and standardised guidance for adapting psychotherapy to the needs of this population. In their previous review, Surley and Dagnan (2019) applied a framework based upon Hurley et al. (1998) to review the frequency and nature of adaptations to CBT when used with people with intellectual disabilities. They demonstrated that most adaptations could be categorised using their framework, but there were issues with coding some adaptations. This was due to a lack of clarity within the literature meaning that some therapy adaptations fell into multiple categories, for example, changing session length could be categorised as simplification or use of flexible methods. There were also issues with potential over‐inclusivity where coded adaptations reflected routine therapy practices rather than specific adaptations made for people with intellectual disabilities. Surley and Dagnan (2019) recommended that researchers need to provide a more detailed description of psychotherapy adaptations.

Our initial framework was based upon the recommendations made by National Institute for Health and Care Excellence (2016), with the aim of enhancing replicability and validity. In summary, National Institute for Health and Care Excellence (2016) recommended that psychotherapy be informed by individual mental health assessments and tailored to individual preferences, include supporters (e.g., parents and carers), and structured to facilitate consolidation of learned skills between sessions. However, the National Institute for Health and Care Excellence (2016) did not thoroughly describe the nature of their suggested adaptations, and there remains a lack of clarity and evidence. To address this, the key aim of our systematic review was to develop a comprehensive framework detailing and describing the nature and frequency of specific adaptations to psychotherapy for use with people with intellectual disabilities. Unlike Surley and Dagnan (2019), our review considered all types of psychotherapies, rather than focusing only upon CBT, with the aim of strengthening the applicability of the final framework to a broader range of therapies. Unsurprisingly, challenges arose with our initial framework (Table S2), similar to those encountered by Surley and Dagnan (2019) as the diverse range, and unclear reports, of adaptations made accurate categorisation challenging. For example, Ashworth et al. (2021) included detailed descriptions of adaptations, such as the use of visual aids that included key images to represent certain topics (e.g., mindfulness), and visual reminders used as references throughout the intervention. They tell the reader exactly what was adapted and their purpose. In contrast, Deb et al. (2022) mention that the use of music therapy, aromatherapy and sensory stimulation may be beneficial for people with intellectual disabilities, but does not provide detailed descriptions of their purpose or implementation during therapy, further emphasising a need for greater detail and explicit reporting in future research. As a result, the framework was expanded to encompass the broader range of adaptations described within the literature. This reduced overlap between categories, providing a more precise and inclusive tool for categorising adaptations.

### 4.1 Nature and Frequency of Adaptations

Within our framework, several common adaptations across different types of psychotherapies for people with intellectual disabilities were characterised. Adaptations involved (1) multisensory methods, (2) activities, (3) communication, (4) delivery medium, (5) additional support and (6) changes to structure. Changing communication (e.g., simplifying language or using prompts) was the most frequent adaptation, followed by changing the delivery medium (e.g., varying the location or incorporating technology). However, this trend could have been influenced by the COVID‐19 pandemic, which may have prompted a shift towards remote delivery and blended approaches. Adaptations to therapy structure (e.g., longer or shorter sessions and increased or decreased sessions frequency) were also frequently described within the literature. Interestingly, incorporating activities (e.g., games) was the least frequently described adaptation. These adaptations, though varied in their application, generally align with attempts to improve the accessibility of therapy with people with intellectual disabilities. Particular attention had been paid to the delivery and structure of psychotherapies, perhaps indicating these to be most adaptable, as portrayed by the breadth of sub‐categories in association. In contrast, additional supporters were not as frequently mentioned within the included studies, which could be due to uncertainties about the roles of additional supporters when adapting standardised psychotherapeutic approaches, as well as logistical and ethical constraints, such as confidentiality concerns or interference with the therapy process (Giacco et al. 2017).

The frequency of the identified adaptations suggests a broad consensus within the field regarding their necessity. All the included studies incorporated at least one intervention adaptation with many having included multiple adaptations. However, the consistency in the application of adaptations varied across the studies. Some adaptations, such as repeating key concepts discussed during sessions to consolidate learning (e.g., Ashworth et al. 2020), were applied consistently due to their structured nature. In contrast, adaptations such as including additional supporters during therapy, varied significantly between studies depending on the therapeutic context (e.g., type of therapy and mental health problem) and individual needs. For example, Charlton and Dykstra (2011) found that involving parents and caregivers enhanced the delivery of DBT, whereas Wilson et al. (2020) incorporated peer mentors to support young adults with intellectual disabilities but found no significant improvement in quality of life or well‐being pre‐ and post‐test. The inconsistent application of adaptations may make it challenging for clinicians to implement these frameworks in practice, as well as complicate comparisons of outcomes across studies. This variability suggests that while adaptations are necessary for individualised psychotherapy, clearer guidelines on their implementation could enhance consistency and better inform clinical practice.

### 4.2 Psychotherapy and Mental Health Conditions

Within our framework, various adaptation categories were commonly applied across different psychotherapies with different mental health problems. This is unsurprising, as the practical and individualised challenges experienced by this group, such as cognitive and communicative difficulties or support needs, often took precedence over diagnostic considerations. While therapeutic techniques and content varied across mental health conditions, many adaptations remained consistent across therapies to accommodate the needs of people with intellectual disabilities. The framework described within our systematic review focused on these shared adaptations, which were implemented to enable effective participation in a range of psychotherapies for people with intellectual disabilities. This approach aligns with a transdiagnostic approach to adaptation, prioritising the most appropriate adaptations to facilitate engagement in therapy, rather than condition‐specific adaptations.

Although CBT, DBT, EMDR and mindfulness were the most noted therapeutic approaches cited within the literature, there were several infrequently mentioned approaches. For example, Loeper and Schwartz (2023) and Schwartz and Levin (2022) examined peer‐mentoring programmes, which were said to be useful for people with intellectual disabilities by improving confidence, emotional well‐being and enhancing coping strategies. Mayer et al. (2023) investigated narrative exposure therapy and reported reductions in PTSD symptoms. O'Riordan et al. (2024) introduced notable adaptations to standardised tools used in complicated grief therapy. Abrego (2020) found that strength‐based therapy improved well‐being, and Newsome Hoyle and McKinney (2015) highlighted the benefits of music therapy with individuals with moderate‐profound intellectual disability. Furthermore, although the review included studies concerned with both adults and children, there was a disproportionally greater number of studies concerned with adults rather than children included in the present review. Future research should be conducted to further understand the adaptations required when conducting therapy with children.

### 4.3 Implications for Future Practice

People with intellectual disabilities are a heterogenous group with varied needs and abilities, which has likely driven the diversity of adaptations identified across the literature. Our findings indicated that the frequency and nature of adaptations may likely vary depending on the severity of intellectual disability, placing particular importance on the use of person‐centred approaches. There are several key adaptations that clinicians need to consider when working with individuals with intellectual disabilities. Firstly, communication barriers are a core challenge in psychotherapy for people with intellectual disabilities. Reducing the reliance upon written and verbal communication using multisensory methods is likely to be beneficial. This would include reducing the complexity of verbal communication by simplifying language, breaking concepts into concrete components and checking comprehension regularly. The use of images, symbols, videos, easier to read materials or audio‐based materials may be beneficial, while for those with more severe intellectual disabilities, therapists may need to make use of specific augmented communication methods such as a Talking Mat (Murphy and Cameron 2008), while working heavily with carers. Secondly, and related to communication, incorporating activities may enhance engagement, improve retention of concepts and offer new ways for clients to express themselves, allowing them to communicate their thoughts and feelings to clinicians more effectively. These activities may include role play, art, games and other physical activities. Thirdly, more directive approaches to psychotherapy, including the use of prompts, repetition and positive reinforcement are likely to be beneficial in maintaining attention and consolidating learning. Fourthly, for some individuals, the inclusion of supporters, such as paid carers, relatives/family members and peers is likely to be beneficial. Supporters can assist with communication, reinforce therapeutic material outside sessions and support homework tasks. The level and nature of supporter involvement should be collaboratively negotiated based on therapeutic goals and client preferences, recognising that supporters are likely to always be involved when working with those with more severe intellectual disabilities. Finally, adaptations to the structure of therapy are likely helpful, which include adjustments to the session length, pacing and frequency along with the utilisation of regular breaks during sessions. Some clients may require shorter, more frequent sessions, while others may benefit from longer sessions. Additional psychoeducation and skills teaching, more frequent progress reviews and more homework may support learning and retention of concepts.

### 4.4 Implication for Future Policy

In the United Kingdom (UK), health services and other organisations are required to ensure equitable service provision for individuals with intellectual disabilities (Brown et al. 2011). However, legislation alone (e.g., Equality Act 2010; Human Rights Act 1998) cannot address the multifaceted needs of people with intellectual disabilities for a variety of reasons including the continuing prioritisation of the needs of the general population, poor development and coordination of effective general healthcare for this group and limited availability of expert providers (Brown et al. 2011). Although a range of adaptations to psychotherapies had been reported within the literature, these were predominantly based upon clinical experience, without clarity as to their development and effectiveness. Therefore, the development of a standardised evidence‐based psychotherapy adaptation framework for use with people with intellectual disabilities would be welcomed. Further research about the effectiveness of such a framework could lead to improved outcomes for individuals with intellectual disabilities. This group is excluded from most clinical trials (Feldman et al. 2014), and they are substantially more likely to experience mental health disorders (Buckles et al. 2013; Mazza et al. 2020) relative to the general population; this represents a ‘double inequality’ that needs to be addressed.

### 4.5 Implication for Future Education

In some countries, training programmes for therapists, psychologists and psychiatrists do not include specific training about working with people with intellectual disabilities (Man et al. 2018). For example, in the United States, there is little to no training for clinicians in working with people with intellectual disabilities (Marrus et al. 2022), which is clearly problematic, and those who do work with this group tend to have either trained under the supervision of someone who is working with this population or developed their expertise postqualification. However, within other nations, such as the United Kingdom, clinical psychologists, psychiatrists, nurses and some allied health professions are required to complete training in working with people with intellectual disabilities (Nisar et al. 2025). This means that incorporating our framework into clinical psychology and therapist training programmes that lack existing training about people with intellectual disabilities may be somewhat more challenging, but nevertheless, the framework may prove to be exceptionally advantageous for such programmes, providing a valuable framework for organising teaching and training, while it is also likely to be beneficial for refining existing programmes.

### 4.6 Strengths and Limitations

One of the strengths of this systematic review is the use of an extensive search incorporating multiple databases, including grey literature such as book chapters and presentations, and using established tools to assess study quality. The data extraction and data synthesis methods enhanced the replicability and validity of the results obtained. However, there are several limitations that must be noted. Firstly, ambiguities in the definitions of intellectual disability made it difficult to judge study eligibility at times. For example, some authors included clear evidence that their participants had intellectual disabilities by reporting the Full‐Scale IQ (e.g., van Wingerden et al. 2021) or included a statement that their participants had either mild, moderate or severe intellectual disabilities, while some did not specify severity at all (e.g., Karatzias et al. 2019; Perera et al. 2022). This also made it difficult to establish whether adaptations to therapy varied depending upon degree of intellectual disability. Secondly, some studies included thorough descriptions of the adaptations made to psychotherapy, including an associated rationale, whereas others did not. At times, this made coding difficult and also made it difficult to understand whether there were variations in adaptability between intervention types, an issue that has also been found in previous reviews (Surley and Dagnan 2019; Witwer et al. 2022). Idusohan‐Moizer et al. (2015) suggested that significant modifications to an intervention may impact the validity of the evidence supporting it, and so, a highly adaptable intervention may not necessarily be most effective. This raises questions regarding the extent to which interventions should be adapted, especially those that were initially developed for use with people who do not have intellectual disabilities. This does increase uncertainty stemming from a lack of standardised guidance for adapting psychotherapies that has been shown to be effective.

While some adaptations, such as the use of visual methods and simplification of language, have been used within RCT (e.g., Willner et al. 2013), there have been no clinical trials testing the actual efficacy of therapy adaptations for actual mental health conditions with people with intellectual disabilities. There have been some trials testing psychotherapy adaptations to determine whether they lead to the acquisition of skills thought necessary to engage in psychotherapy (Bruce et al. 2010; Vereenooghe et al. 2015, 2016); whether these adaptations improve outcomes from actual psychotherapy has not been determined. Most therapy adaptations described in the literature are based upon clinical experience, a finding that is consistent with previous research on the topic (e.g., Dagnan et al. 2018; Witwer et al. 2022). This is evidenced by the large proportion of single‐arm within‐subjects designs (*n* = 26) and descriptive case study designs (*n* = 15) included within the current review. This emphasises a need for more rigorous studies testing adaptations to understand the extent to which adaptations to psychotherapies are effective. Furthermore, the relatively small sample sizes within included studies, including RCTs, threaten the validity of the conclusions reached within these studies and add further complexity when attempting to ascertain the efficacy of adapted psychotherapies (Tapp et al. 2023; Vereenooghe and Langdon 2013).

There were also inconsistencies in the reporting of participant information, intervention implementation and adaptations, which contributed to uncertainties about the most relevant articles for inclusion. It is important for future studies to be consistent with their definitions, as well as with the reporting of information, to avoid misinterpretations. Our findings also emphasised the need for larger clinical trials with standardised methods to improve the quality of data available within the literature.

Overall, the quality of included studies, as assessed using the MMAT and JBI case‐series checklists, were within the moderate‐to‐high range, indicating that most of the studies demonstrated clear research questions, representative participant samples and appropriate methodologies. However, several methodological limitations were evident, particularly in studies with lower quality ratings. For example, incomplete outcome data, as observed in studies such as Wilson et al. (2020) and Verhagen et al. (2023), raised concerns about the reliability of findings while little can be said about whether the adaptations used were effective. Similarly, studies with limited sample diversity, such as Ashworth et al. (2021), which exclusively included male participants, highlight issues of representativeness, potentially limiting the generalisability of results. Furthermore, studies like Jones et al. (2021) struggled to clearly integrate qualitative and quantitative results, reducing the coherence of their findings and the extent to which they inform practice. Studies assessed using the JBI case‐series checklist similarly demonstrated moderate‐to‐high quality overall, with most providing adequate clinical information and clear reporting of outcomes. However, Abrego (2020), which received a score of two, exemplified how insufficient detail regarding participant demographic characteristics, history, current clinical condition, assessment methods, description of treatment procedures and adverse/unanticipated events can hinder transparency and replicability of research. These findings highlight the need for improved methodological rigour in future studies, including more comprehensive reporting of data, diverse and representative samples and clearer integration of mixed‐methods approaches. Addressing these issues may enhance the quality and reliability of evidence, ensuring greater applicability to clinical practice while allowing for an improved understanding of whether adaptations to psychotherapy and adapted psychotherapy are efficacious. Looking forward, as adaptations to therapy are likely to vary according to the presenting problem and severity of intellectual disability, we would suggest that researchers focus upon determining whether identified adaptations to therapy lead to improved outcomes, and their associated mechanism of change.

## 5 Conclusion

Individuals with intellectual disabilities are a heterogenous group, and it is important for clinicians to consider the needs of individuals to understand how to appropriately adapt psychotherapy in a person‐centred manner. The present review aimed to provide a comprehensive review of the nature and frequency of adaptations that have been made to psychotherapies to accommodate the needs of individuals with intellectual disabilities. Information present within this review could aid in the development of standardised guidance for conducting therapy with individuals with intellectual disabilities by highlighting the key considerations that should be made when adapting psychotherapy for this population. Further research needs to be conducted to better understand the efficacy of adapted psychotherapies, as well as how severity of intellectual disability may impact the effectiveness of adaptations. Further research is also required to identify the most appropriate adaptations based on intervention type. Emphasis is therefore placed on the need for larger randomised studies to be conducted using standardised measures that enhance replicability.

## Author Contributions

Literature searches and study eligibility was conducted independently by A.C.P. and O.H. Data extraction of included studies was conducted independently by A.C.P. and O.H. Quality appraisal for included studies was conducted independently by A.C.P. and O.H. The first draft of the manuscript was written by A.C.P., and all authors contributed to and approved the final manuscript. The process was supervised by P.E.L. and K.M.G.

## Funding

The project was funded by a University of Warwick Collaborative Fellowship (WCF) scheme in collaboration with Herefordshire and Worcestershire Health and Care NHS Trust and formed part of a PhD completed by A.P. The funder had no role in the study design, collection, analysis or interpretation of the data, writing the manuscript or the decision to submit the paper for publication.

## Conflicts of Interest

The authors declare no conflicts of interest.

## Supporting information


**Table S1:** Data extraction table.
**Table S2:** An initial framework for adapting psychological interventions that was developed using NICE (2016) guideline.
**Table S3:** Final framework.

## Data Availability

All data used in the present systematic review can be found in the Supporting Information.
