# Supplementary material for: Adapting Psychological Therapies for Individuals With Intellectual Disabilities: A Systematic Review
Source: Clin Psychol Psychother. 2026 Jan 7;33(1):e70202. doi: 10.1002/cpp.70202 (PMC12775901; doi:10.1002/cpp.70202)
Supplement: Supplementary file 1 — Table S1: Data extraction table. Table S2: An initial framework for adapting psychological interventions that was developed using NICE (2016) guideline. Table S3: Final framework. [file CPP-33-e70202-s001.docx]

**Supplementary materials**

**Search Terms**

***Intellectual disabilities***

(intellectual* adj1 disab*).ab,ti.

(learning adj1 disab*).ab,ti.

(intellectual* adj1 deficien*).ab,ti.

(intellectual* adj retard*).ab,ti.

(intellectual* adj1 impair*).ab,ti.

(intellectual* adj1 handicap*).ab,ti.

(intellectual* adj1 subnormal*).ab,ti.

(learning adj1 difficult*).ab,ti.

(learning adj1 deficien*).ab,ti.

(learning adj1 impair*).ab,ti.

(learning adj1 retard*).ab,ti.

(learning adj1 subnormal*).ab,ti.

(learning adj1 handicap*).ab,ti.

(mental* adj1 retard*).ab,ti.

(mental* adj1 deficien*).ab,ti.

(mental* adj1 handicap*).ab,ti.

(mental* adj1 disab*).ab,ti.

(mental* adj1 impair*).ab,ti.

(mental* adj1 subnormal*).ab,ti.

(intellectual adj1 developmental adj1 disab*).ab,ti.

(intellectual adj1 development adj1 disorder*).ab,ti.

(down* adj1 syndrome).ab,ti.

(fragile adj1 x adj1 syndrome).ab,ti.

(fragile adj1 x).ab,ti.

(william adj1 syndrome).ab,ti.

(angelman adj1 syndrome).ab,ti.

(profound* adj2 multiple adj1 learning adj1 disab*).ab,ti.

(profound* adj1 intellectual* adj1 multiple adj1 disab*).ab,ti.

(Rett adj1 syndrome).ab,ti.

(overgrowth adj syndrome).ab,ti.

(Asperger* adj1 syndrome).ab,ti.

(autis* adj1 spectrum adj1 disorder).ab,ti.

(pervasive adj1 developmental* adj1 disorder*).ab,ti.

(pervasive adj1 developmental* adj1 disorder* adj2 otherwise specified).ab,ti.

(f?etal adj1 alcohol adj1 syndrome).ab,ti.

(f?etal adj1 alcohol).ab,ti.

(prenatal adj1 alcohol adj1 exposure).ab,ti.

(velocardiofacial adj1 syndrome).ab,ti.

(klinefelter adj1 syndrome).ab,ti.

(childhood adj1 disintegrative adj1 disorder).ab,ti.

(smith adj1 magenis).ab,ti.

(cri adj1 du adj1 chat).ab,ti.

(cornelia adj1 de adj1 lange).ab,ti.

(de adj1 lange).ab,ti.

(genetic adj1 disorder*).ab,ti.

(static adj1 encephalopathy).ab,ti.

(complex adj1 need*).ab,ti.

(special adj1 education* adj1 need*).ab,ti.

(special adj1 need*).ab,kw,sh,ti.

(special adj1 education* adj1 need* adj2 disabilit*).ab,ti.

(special adj1 need* adj2 disabilit*).ab,kw,sh,ti.

(developmental* adj1 disab*).ab,kw,sh,ti.

(neurodevelopmental* adj1 disorder*).ab,ti.

(developmental* adj1 disorder*).ab,ti.

(neurodevelopmental* adj1 disab*).ab,ti.

(neurodevelopmental* adj1 impair*).ab,ti.

(neurodevelopmental* adj1 retard*).ab,ti.

(neurodevelopmental* adj1 handicap*).ab,ti.

(neurodevelopmental* adj1 subnormal*).ab,ti.

(neurodevelopmental* adj1 deficien*).ab,ti.

(developmental* adj1 delay*).ab,ti.

(developmental* adj1 difficult*).ab,ti.

(developmental* adj1 impair*).ab,ti.

(developmental* adj1 handicap*).ab,ti.

(developmental* adj1 retard*).ab,ti.

(developmental* adj1 subnormal*).ab,ti.

(abnormal* adj1 develop*).ab,ti.

(prader adj1 willi adj1 syndrome).ab,ti.

***Psychological Therapy***

psycho* adj2 (therap* OR treatment* OR intervention*)
training OR management OR counsel*
psychotherap*

***Mental Health***

Mental health OR Mental Disord* OR Mental Illness

Anx*

Depress*

Anger

Mood

Trauma*

Phobia

Social

Self-concept

Emotion* disord*

Determination

Bereave*

Disord*

Hyper*

Attent*

Mental problem*

Substance abuse

Substance

No restrictions on publication dates or geographical location were applied. Only articles written in English were included.

**Table S1**

*Data extraction table.*

| **First Author (Year)** | **Study type** | **Participants** | **Intervention** | **Adaptation** | **Future adaptation recommendations** | | **Outcome** | **Follow up** | | **Quality of paper (MMAT)** | **Quality of paper**  **(JBi case series)** | |
| --- | --- | --- | --- | --- | --- | --- | --- | --- | --- | --- | --- | --- |
| 1. Datlen (2020) | Journal article Qualitative  Descriptive | N=5 Age: 23-27 years Intellectual disability: (unspecified) | Online modality Visual methods- Art-based therapy: - Colours used to draw and paint. - Colourful clay sculptures used  - These methods used to express emotions. Using WhatsApp allowed for text, sound, images, videos and emojis to be utilised whilst communicating with clients. | Online modality. Personalised art materials (All supplied a sketchbook and had options of 2 other options of choice). | Prioritise therapeutic relationship over feedback. | | Group was reported to be useful for 4/5 clients. However, although one client reported not enjoying it, they still used art to express their emotions in their life. | N/A | | N/A | 5 | |
| 2. Campbell and Scarpa (2019) | Book chapter Psychotherapy for developmental disabilities | N/A | N/A | Visual supports Brief, clear language - reduce abstract language. Increase repetition - consolidate learning. Use a slower pace - include breaks, divide sessions. Involve caregivers and direct care staff - to consolidate gains in therapy and assist with therapeutic homework. | Interdisciplinary training - to meet various individual needs of individuals. | | | | | | | |
| 3. Didden et al. (2019) | Book chapter Treatment of anger and violence in individuals with intellectual disabilities | Intellectual disabilities (unspecified)  Anger and violence | CBT | Use shorter sentences containing a single concept. Use words fewer than three syllables. Ask clients to summarise the session in order to assess their understanding and retention. Use inductive methods (e.g. socratic dialogue). Use role-play. Increase motivation to change. Work with significant others and relatives. Skills and prompts: Recognise emotions by using photographs of people's faces. Discriminate between thoughts, emotions, and behaviour by using personally relevant scenarios and pictorial stimuli. Make connections between events and emotions, and recognising the mediating role of thoughts in the relationship between thoughts, emotions and behaviour by using examples of situations and emotions and behaviours that are congruent.  Adaptations for children:  Attention: use shorter, more frequent sessions reduce task length (smaller units), prevent distractions. Working memory: use memory aids (e.g. visual prompts), present one task at a time, use short, simple, subject-verb object sentences.  Executive functions: use structured sessions (e.g. visual schedule), minimise switching between tasks, redirect uninhabited responses. | Focus more on therapy ending with individuals with intellectual disabilities. Extend follow-up period. Slower-paced therapy to allow more times for P to practice skills. | | | | | | | |
| 4. Wilson et al. (2020) | Journal article.  Quasi-experimental single-group pre-post design | N= 18 Mean age: 19.6 years Intellectual disability: mild-moderate Mentors: Median age: 74 years  Depression/Anxiety | 6-month intervention. Mentees were paired with a mentor that also had a back-up mentor to cover any primary mentor absence from the Men's Shed (community-based organisations). An occupational therapist (OT) was charged with providing 1:1 support to mentor-mentee dyad. OT used targeted and individual support to mentor in how to promote mentee’s activity and social participation, while simultaneously building meaningful relationships between mentor and mentee over time. This support was reduced after 6 weeks. | Inclusion of mentors to support participants with intellectual disabilities. | N/A | No significant difference in well-being and quality-of-life scores (material health, productivity, intimacy, safety and emotional domains) pre to post test. Communication difficulties between mentors and mentees reported. | | | N/A | 3 | N/A | |
| 5. Ashworth et al. (2020) | Journal article. Pre-post intervention Case-study. | Female patient aged 23 years with diagnosis of intellectual disability and ASD Intellectual disability: Mild (IQ: 60) Mental condition:  Emotional dysregulation and instability of mood including depression-like symptoms. | One-hour individualised CBT delivered weekly (18 sessions in total) | Take into account individual needs. Deliver therapy in a structured and concrete manner. Use of psychoeducation regarding thoughts, feelings and behaviour. Functional analysis - used to examine causes and consequences of behaviours – adapted for people with intellectual disabilities by exploring incidents utilising an antecedent, behaviour, consequences (ABC) approach allowing for structure and repetition. Simplified language - easy-read materials - one side contained information regarding ASD - Other side listed information regarding how P felt these traits related to her - allowing her to express these thoughts and feelings in her own words. - This was reviewed by a language and speech therapist.  Visual aids (e.e. 'my autism' sheet, diagrammatic formulation). Use of role-play. | Focus more upon therapy ending with individuals with intellectual disabilities. Extend follow-up period. Slower-paced therapy to allow more times for P to practice skills. | Improvement in self-reported depression symptoms during intervention; however increased symptoms of depression reported towards the end of intervention (end of phase 6). | | | Weeks 19-22. -Increased anxiety and depression scores | N/A | 8 | |
| 6. van Wingerden et al. (2021) | Multiple-Baseline Case study design.  Mixed-methods. | N=10 Mean age: 46.90 years Intellectual disability: Moderate (IQ 40-50)  Anxiety | A robot was used as a conversational partner.  3 weekly sessions Setting: Vocational centre, and participants' home (2 participants) | Robot-mediated intervention. Feedback obtained from caregivers and researchers with people with intellectual disabilities in order to adapt robot functioning and scripts to take into account individual needs of clients and relate to participants’ level of functioning and life experiences: - Tone of voice altered to be as natural as possible.  - Slowed speech to give participants enough time to process words. - Intonation added to further promote comprehension. - Robot was brightly coloured and placed in front of contrasting background. Gestures during speech were predominantly wide arm movements - Bright LED lights in eyes turned different colours to amplify emotions. - Robot did not move around so that participants did not have to track its location during the conversation - Robot conveyed empathy by giving a short introduction for participants to get used to its voice and reassured participants that it was also 'nervous as it had not met many humans yet'.  - Introduction also served secondary purpose of making participants be more lenient towards any mistakes or incongruent responses the robot might make. To help participants memorise three coping strategies: - Each strategy was related to a tactile object. - Three objects were on the table during each session. - Each of the three conversations had an identical structure to allow participants to internalise the flow of conversation. - Three coping strategies were explicitly repeated at several pointes in conversations. -At the beginning and end of each session, participants were asked to explain strategies. | Three common coping strategies for diverse population may be too generic  - Have several variations of each type of exercise, enabling a personal fit to be made for each individual. Focus more on participants’ personal problems and use personal experiences to practice coping strategies. Involve parents or caregivers to help participants apply these strategies in daily life. | No significant difference in worrying pre and post intervention | | | No follow up | 4 | N/A | |
| 7. Abrego (2020) | Dissertation Client perspective of the experience and impact of participating in strengths-based therapy Case study Qualitative | N=1 Age: 18+  Intellectual disabilities: unspecified | 5 sessions of strengths-based therapy | Adapting language - simplify concepts and sentences to suit client needs. - Individualised psychotherapy approach - Adapt language with written information | N/A | Improved well-being, improved skill with coping with anger and skill in regulating anger. | | | N/A | N/A | 2 | |
| 8. Komarek (2020) | Doctoral thesis  Single-arm - within subjects pre-test/post-test design Mixed-methods | N=5 Age: 21+ years Intellectual disabilities: Moderate-severe Comorbid mental illness - including schizophrenia, PTSD, Trichotillomania, Kleptomania, General Anxiety Disorder, Intermittent explosive disorder. | 10-15 sessions of sandtray therapy | Use of creative, expressive modalities for therapy (sandtray). Therapist generally remained a quiet observer - only attending to sandtray creations empathically and nonverbally. Positive affirmation used throughout process - compliment clients' work. participants’ level of development considered during construction phase - e.g. tracking participants play as done in child-centred play therapy. Simplified language - metaphors used in sessions when verbally processing participants’ work - makes descriptions less personal/intrusive. | Therapists should be open to a variety of therapeutic interventions when working with intellectual disabilities adults (including sandtray). Prioritise a safe space that encourages the development of a meaningful therapeutic alliance. | Positive well-being significantly improved. | | | N/A | 4 | N/A | |
| 9.Whitehead et al. (2021) | Journal article.  Mixed-methods. Study 1: Quantitative longitudinal study measuring changes in young people's behavioural and social issues, mental health, and levels of impairment from acceptance to the service until completion. Study 2: Qualitative interviews with parents/carers who attended the service | Study 1: N=67 Mean age: 15.56 years Intellectual disabilities:  -Severe (3%) -Mild (24%) -Moderate (52%) -Borderline (4%) -Unspecified (16%) Participants had a range of mental health diagnoses Study 2: N=10 (parents/carers) | Program comprises screening, assessment, treatment and consultation for improvement of mental health, physical health and well being in people aged 12-25 years  Two service pathways:  1) Consultation and short-term assessment and intervention - Up to 12 weeks   2) Comprehensive assessment and management program - Over 12 weeks  Psychological therapies offered were Cognitive Behavioural Therapy (CBT), Dialectic Behaviour Therapy (DBT) and Family Therapy. Setting: Homes, schools, workplaces or community | Inclusion of carers ('key worker' to provide central support and contact for the family, integration of interdisciplinary input and follows up on treatment plan goals).  Using preferred style of communication and tailoring mental health treatment to meet the young participants’ intellectual capacity. Multidisciplinary approach - collaborations between clinicians, parents and carers . Emphasis placed on flexibility of treatment approach (e.g. adapt meeting times, methods and communication with parents, and parents not having to attend every session) - reduced stress in parents. | Improve staffing consistency. | Improvement with social problems and behaviour in participants with intellectual disabilities; significant reduction in Health of the National Outcome Scale for child and adolescents (HoNOS-CA) scores. Parents felt supported by the program and service provided was improvement to previous service experiences. | | | N/A | 4 | N/A | |
| 10. Digman (2021) | Journal article Case study  Qualitative | N=2 Age: Early 20s  Intellectual disability: Moderate PTSD | Methods from Psychological first aid, systemic therapy, narrative therapy and interpersonal psychotherapy used.  Therapeutic interventions were used periodically over 2 years post disclosures. Each intervention lasted between 6 and 20 weeks. | Multiple therapeutic approaches used. Inclusion of carers. Drawings used to enhance explanations. Language kept consistent and clear - speech and language assessments were used to inform this practice. Use of role-play - Repetition of validation of feelings to accommodate for triggers that arose after new disclosures.  Emotional expression and pictures used to limit reliance on verbal communication. Worry doll - used as coping mechanism. Reassurance and rapport building: - Sitting outdoors with tea and cake or walking together. - Mirroring expression and mood then structuring responses to these, so moving the 'conversation' nonverbally. - Creating a therapy routine that could gradually and safely explore difficult emotions. | N/A | P1: Decrease in episodes of distress from 3-18 months post-disclosure. Emotional distressed still reported as extreme at 18 months but frequency decreased. Coping mechanisms learned. Engagement in regular day service activities at 2 years, with communication tablet being gradually introduced. P2: | | | N/A | N/A | 7 | |
| 11. Croom et al. (2021) | Journal article  Qualitative  single-arm  Semi-structured interviews | N=9 Mean age= 34.10 years ID: Mild-moderate Anxiety, depression, eating disorder, anger  Country: UK | Two mindfulness groups delivered consecutively aimed to support management of anxiety, low-mood or anger.  There were 8 sessions over 8 weeks, each session lasted 1.5 hrs.  setting: Vocational centre | Simplified language - easy-read adapted documents Supporting explanations with pictures and shared drawing activities. - Use of verbal and non-verbal communication. Skills-based/experiential exercises. Shortened length of exercises. Modelling of activities. Large printed picture of thermometer for checking in/checking out exercise. Repetition to aid assimilation and learning. Psychoeducation. | N/A | Participants found experience of group meaningful and enjoyable.  Benefits included: feelings of self-determination, friendship, support, socialisation, acceptance and sharing. | | | No follow up | 5 | N/A | |
| 12. Rawlings et al. (2021) | Journal article Videoconference-mediated psychological therapy  Single-arm Qualitative | N=22 Age: 18-57 years Intellectual disability: Mild-moderate  Anxiety, depression, anger, bereavement, feeling disconnected from others | Videoconference-mediated psychological therapy | Audio and visual methods (telephone and video call). Simplified language and shorter sentences. Educational materials (leaflets, drawings and worksheets via post) | Identify most appropriate computer program/app to use - ensure it is accessible to people with intellectual disabilities. - Be careful using apps that review personal details such as phone numbers (WhatsApp), ensure appropriate boundaries are in place prior to therapy. Therapists should plan sessions in advance to ensure appropriate physical materials reach clients by post. Use multi-sensory approach and simplify language/materials/concepts with shorter sessions. Include family members/carers to help aid in technology use. Therapists should develop contingency plans with clients and caregivers in the event of problems arising due to connectivity, or clients ending calls abruptly. Repetition within and between sessions. Personalised approach - support clients with intellectual disabilities in making decisions regarding pacing, timing and content of therapy. Structured approaches are helpful, but a flexible approach could improve adherence. | Majority of clients unable to engage in video-conference, and were only suitable for phone therapy. One felt safer conducting therapy over the phone due to coronavirus. Four clients mentioned reduced waiting time for treatment. | | | N/A | 5 | N/A | |
| 13. Newlands and Benuto (2020) | Journal article.  Case-study. Qualitative. | N=1 Age: 37 years Intellectual disability: Indications of mild intellectual disability (IQ=63) Depressed mood, anxiety | 11 weekly sessions of DBT, which included psychoeducation, emotion regulation, interpersonal effectiveness, distress tolerance, mindfulness and validation. Included additional week for review. | Individual instead of group setting (extra attention and rehearsal). More time reviewing homework and skills from previous weeks. Simplified topics and language. Streamlined handouts. Additional sessions on interpersonal/relationship effectiveness. Every session interlaced topic of validation into discussion.  Frequent check-ins of client understanding of content. Reduced number of skills covered per group (1-3 rather than 3+, more time allocated per skill, systematic approach). Behavioural chain conducted around situations, extra time spent with client generating possible issues that may hinder use of skills.  Language and topics simplified. Streamlined Handouts. Additional sessions spent on interpersonal/relationship effectiveness. Every session interlaced the topic of validation into discussion. | Individual rather than group format. Group format with less material per session may result in clinically significant improvements and allow clients to interact with peers. However, care must be taken to give clients the right amount of attention. Language and presentation of handouts, worksheets, and diary cards should be modified to ensure information easier to understand and retain. | Decreased depressive symptoms, but increased fatigue - speculated to be due to increased caffein intake (12 sodas a day) | | | N/A | N/A | 8 | |
| 14. Verberg et al. (2022) | Journal article.  RCT Two-arms:  EG: TGF CG: TAU Quantitative. | N=119 Age: 12-23 years Intellectual disability: Mild-borderline (IQ: 50-85) Comorbid problems including physical disability and psychiatric disorder | 6 sessions of online "The growth factory", each session lasting 25-40 min.  Improve confidence and self-esteem - includes education about plasticity of brain and ability to change Setting: Vocational centre | Modality - online. Visual and auditory methods (game-like approach, videos). Inclusion of avatars to teach concepts, and act as 'role-models' in video clips - Included stories and struggles that matched those often reported by youth with intellectual disabilities. Participants were able to choose their own topic in assignments (flexible approach | N/A | TGF shown to be effective in increasing perseverance and self-esteem, in decreasing internalising, attention and total mental health problems, and in maintaining participants' collaboration with their mentor immediately after the intervention.  No significant differences between the intervention and control group in mindset of emotion and behaviour and mindset of intelligence. | | | 3 to 6 month follow-up study effects maintained | 4 | N/A | |
| 15. Ashworth et al. (2021) | Journal article. Single case study. Qualitative. | N= 5 Age= 23-57 years Intellectual disability: mild Comorbid psychiatric diagnoses including mental illness, ASD and personality disorder | Second edition of adapted DBT informed group based-skills programme administered. Program duration: 14 months | Minimal inclusion of written material. Literacy was simple and short in participant handouts. Visual aids used - included key images to represent certain topics (e.g. mindfulness) and repeated throughout the programme to increase recognition. Visual reminders developed for display purposes to create a total communication environment used as references throughout the group - in addition to non-written alternative activities and practice tasks suggested (e.g. behaviour 'experiments' etc). Concept of procedural learning or behavioural rehearsal was used, and suggestions were given to stimulate learning via non-traditional means to support facilitators.  Positive reinforcement using a variety of methods- positive reinforcement schedules, demonstration of progress (e.g. tick chart of sessions attended), tangible rewards (e.g. certificates at the end of each module), facilitator and/or peer acknowledgement of positive achievements. Additional resources included (e.g. scripts, mindfulness activities, skill flashcards).  Updated artwork to increase inclusivity and relevance to different groups. Pictorial diary card added to facilitate individual based work for DBT trained clinicians to wish to extend the clinical utility of the programme for their clients. | N/A | Increased people skills post intervention. No significant difference between pre and post mindfulness module scores. No significant difference pre and post managing feelings module scores. No significant difference pre and post coping in crisis module scores. No significant difference pre and post-therapeutic factors scores. | | | N/A | 3 | N/A | |
| 16. Gregson and Delaney (2021) | Journal article. Single case study. Qualitative. | N=1 Age: In her 50's Intellectual disability: unspecified severity Trauma, loss | Trauma informed care within a systemic team formulation during COVID-19. | Face-to-face. Systemic approach to treatment - working with Participant’s care team in order to support the system.  Team viewed themselves as part of the system as opposed to separate from Participant. | N/A | Staff found the session helpful.  Mapped out patterns and behaviours in feedback loop helped staff better understand the client. | | | N/A | 5 | N/A | |
| 17. Hollins and Sinason (2021) | Book chapter. "Therapeutic approaches to abuse and intellectual disability: the invisible victims" | Intellectual disability Trauma | Psychotherapy and counselling Both group and individual | Visual methods (Pictures) - In colour - Used for education - Used to explain concepts Simplified/removal of written language  Inclusion of carers | N/A | N/A | | | N/A | N/A | N/A | |
| 18. Jones (2021) | Journal Article. Pilot RCT. Two independent groups: EG: DBT (aDBT-ID) CG: Treatment as usual Mixed methods. | N=20 (total) EG: N=10 CG: N-10 Age: 22-52 years Intellectual disability: Mild-moderate BPD, anxiety, depression, Trauma, SAD | EG: 2hr sessions over 12 week period Includes homework, involvement of carers, mindfulness, core concepts and novel skills. Semi-structured Interviews held for group experiences and updates. Group size: 10 Setting: Vocational setting Face-to-face | Inclusion of caregivers Integration of mindfulness Visual methods (pictorial materials, visual representation of concepts). Simplified language Repetition for skill retention. Consolidation of sessions with integrated caregiver coaching. Reduced session dosage to improve retention. Accessible community crisis team for 24hr support (Delivery of wider DBT programme). | N/A | Positive response from both caregivers and people with Intellectual disabilities. | | | N/A | 5 | N/A | |
| 19. Ugwu et al. (2022) | Journal article. RCT. Two arms: EG: Rational emotive behaviour therapy CG: Treatment as usual  Quantitative. | N=48 Age: >5 years Intellectual disability: Unspecified severity, however, low score on the Wide Range Achievement Test (WRAT) was an inclusion criteria for the study. Depression | 2 sessions of REBT per week, each session lasted 40 min. 12 weeks total. 3 modules: 1. Influence of participants' thoughts on their mood 2. Influence of participants' activities on their mood 3. Influence of participants' relationships on their mood | Integrated music into every aspect of CBT group therapy - to enhance comprehension and engagement with material (This includes critical listening to musical material, songwriting, playing instruments and using music as a point of reference in group discussions as well as homework). | Accurate diagnoses of LD. | Greater decrease in depressive symptoms in EG compared to CG | | | 3-month follow-up, study effects maintained. | 4 | N/A | |
| 20. Schwartz and Levin (2022) | Journal article.  Single-arm.  Mixed-methods. | N=5 Age: 19-23 years Intellectual disability: unspecified severity  2 Had autism Depression, anxiety, Co-occurring mental health conditions  Country: USA | Peer mentors and participants met weekly for 10 weeks. Sessions lasted 60-90min. Aimed to increase use of two coping strategies, and identify one community0based coping strategy or activity.  Setting: Personal choice of participants (e.g. schools, homes, libraries, coffee shops etc) | Inclusion of peer-mentors with intellectual disabilities. Flexible settings where peer-mentors and participants met. Mentor's followed flexible scripts for psychoeducation and activities to support exploration of coping strategies - allowed for tailoring to suit individual needs of participants. Several activities and worksheets given to P's to support attainment of peer mentoring objectives. Visual methods (Videos about mental health). Role-play for mentors to practice sessions and individualise mentoring scripts - became familiar with content and activities. Inclusion of parents | Increase time for socialisation. Flexible content. Increased communication with participants' supporters. Reduced use of script to structure mentoring sessions. | Feasibility testing suggests peer mentoring to be a potentially feasible approach to support young adults with IDD and co-occurring mental health conditions to identify and utilise strategies to cope with mental health symptoms. | | | N/A | 5 | N/A | |
| 21. Vereenooghe et al. (2021) | Journal article.  Descriptive  Qualitative. | N=12 Mean age: 43.75 years Intellectual disability: unspecified  Depression | Online mental health intervention.  Participants were exposed to one of two websites: 1) Moodgym 2) iFightDepression | Online modality. Visual methods: - Participants identified with images that had personal connection with them (e.g. riding a bike). - Realistic pictures preferred over generic drawings or symbols when illustrating emotions. - Pictorial symbols useful to support users in understanding text.  - Graphical elements (e.g. arrows) to show connections between thoughts, feelings and interactions. - Use of diagrams with colour coding - helps with interpreting diagrams. - Mathematical symbols (e.g. plus and minus) support understanding. Easy-read texts - bold words, reduced amount of text used. Auditory support: - Text better understood when read out loud. Online coloured feedback after clicking - help participants navigate the website. | Include alternative media, such as videos. Modify sample characters involved in problem - high level of emotional investment when exposed to problems of sample characters, some found demonstrative tone of program difficult to grasp. More characters to be included with less exaggeration in character tone and behaviours.  Include summary of program - aid in memory when logging back into website. | Overall, website was user-friendly, however, not considered a replacement to personal interventions. Further modifications required. | | | N/A | 5 | N/A | |
| 22. Blakeley-Smith et al. (2021) | Single intervention group.  Family-focused CBT intervention. Quantitative. | N=23 Age= Between 12 and 19 years Intellectual disability: moderate (IQ=58.3) Mental condition: -Anxiety | 45-60 min Family-focused CBT group intervention.  Group size: 2-4 families | Reduced session length from 90 min to 45-60 min. Reduced size of group from 4-6 families, to 2-4. Parents and teens participated together in dyads. Parent curriculum front loaded onto the programme (3 parent sessions preceded the 11 parent-teen dyadic work sessions with a breakout parent/teen session taking place at session 7). Visual structure (Pictorial-based instruction, video modelling). Repetition. Hands on activities to learn anxiety management. High frequency reinforcement of brave behaviour. Tailored support for problem behaviour  Somatic management (labelled 'Calm body'- included visual menu of strategies such as deep breathing and sensory regulation techniques). | More tailored tracking, assessment and treatment of problem behaviour. | Reduction in anxiety symptoms. Significant reductions noted in all subscales (ADAMS, SCARED-P, FSSC-R). | | | 6 week follow up. Parents | 4 | N/A | |
| 23. Weber and Streicher (2021) | Book chapter. | Older individuals with intellectual disabilities. | | Therapists should use plain syntax when addressing clients. Initiating communication may take longer (several sessions required to develop a relationship between therapist and client). Greater consideration of non-verbal communication signs (e.g. quality of eye contact, gaze expression). Use simple examples that are understandable and meaningful when explaining things to the client or enquiring. Rehearsal - a directive approach to be used by the therapist in order to re-inform client about reasons for therapy attendance, as well as the objectives of therapy plan.  Address client according to their perceptive and intellectual grasp. Point to rules of therapy and stick to them in order to ensure client adheres to the intended therapeutic process. - Therapist could point to limits and inappropriateness of certain behaviours that hinder therapeutic process during sessions, and recognise client's current emotional need as well as elaborate with client on alternative behaviour.  Directive approach adopted for potential lack of self-initiative in communication.  Greater flexibility in techniques, methods and settings. Length and frequency of sessions are largely determined by adult's endurance and attention.  - Psychotherapeutic sessions for people with Intellectual disabilities shorter and more compactly scheduled compared to those without. - Older people with Intellectual disabilites: 20-25 min sessions twice a week may be enough.  Use third-party informants (e.g. family members, staff or other caregivers). Consider cognitive functioning of client when determining and selecting therapeutic approach. - Some methods require good short-term functioning. - Groups used in group therapy should be homogeneous with respect to the level of cognitive functioning and communicative skills (more capable adults may feel they are not being taken seriously) - Digital and smart communication technologies can increase accessibility of psychological therapy. Behaviourally-based therapies deployed in conjunction with 'simulated presence therapy', and/or 'individualised music therapy', can improve efficacy. Individualised music therapy: - Client listens to preferred pieces of music Simulated presence therapy: - Client listens to prepared audio sequences which offer selected segments of their memory known for calming and comforting effects on the individual. Music therapy and chair yoga shown to have positive effects on the moods of p's with dementia, as well as reduce risk of falls.  Prouty's pre therapy - therapist sets up contact by repeating gestures, vocalisations and body postures of the adult and offering reflections. - Aims to restore or develop a client's contact functions (contact to reality, affective contact or communicative contact). Use of relaxation techniques (e.g. muscle relaxation for anxiety). Explore areas of activities meaningful to the person prior to disease or functional loss. | N/A | N/A | | | N/A | N/A | N/A | |
| 24. Singh et al. (2021) | Book chapter. | N/A | Mindfulness care giving support for anger and aggression management | Mindfulness-Based Positive Behaviour Support (MBPBS) - consists of two components: 1. Mindfulness 2. Positive Behaviour Support (PBS) Inclusion of parents/caregivers. Use of teachers within mindfulness-based programs and practices for anger and aggression in school settings. | N/A | N/A | | | N/A | N/A | N/A | |
| 25. Keesler et al. (2023) | Journal article. Case series. Qualitative. | N=4 Age: 22-41 years Intellectual disability: Three had Mild (IQ: 50-70) One had Autism  Trauma | Progressive Counting (PC) implemented within the context of Fairy Tale Model (FTM) - based on motivational interviewing. Therapist read around the fairytale, shared corresponding illustrations and contextualised the trauma of participants by pairing their personal experiences with the components of the fairytale. 7-9 sessions of PC , each session lasted approximately 1hr Setting: Outpatient clinic | Visual methods - use of an illustrated framework, visual prompts (pictures and movie reels) |  | Improvement in trauma-related symptoms, including behavioural problems. PC well tolerated | | | N/A |  | 5 | |
| 26. Browne and Smith (2016) | Doctoral Thesis. semi-structured interviews.  Qualitative. | N=9 Mean age: 30.3 years Intellectual disability: unspecified severity  Anger, aggression | Dialectical Behaviour Therapy (DBT) in forensic intellectual disability services. | Jargon-free, simplifying language and short sentences containing single concepts. Indicative and experiential methods to illustrate concepts and promote learning, including: -Pictorial and audio stimuli -Socratic questioning -Role-play Frequent repetition and recapitulation of skills to facilitate retention. Flexible sessions - tailor therapy to individual needs. Reformatting written materials rehearsal of skills and assistance outside of sessions with homework. | N/A | Core category - Uphill and downhill journey of skill use - Explains participants engagement with DBT and set within wider context of three supporting categories: 1. Extrinsic compliance  - Focussed on individual's motivation to commence DBT 2. Sense of safety - Individuals' perceived vulnerability over course of programme  3. Belief in self - Shift of individuals' sense of capability to learn and utilise new information and skills over course of programme | | | N/A | 5 | N/A | |
| 27. Fabian et al. (2024) | Two-arm non-randomised pilot study. Quantitative | Total sample N=24  Group 1 (more support): Mean age: 38 years (SD=8.52) Group 2: Mean age: 38.5 years (SD=10.98)  Sub-sample N=12 (appropriateness) Group 1: Mean age: 35.33 years (SD=6.12) Group 2: Mean age: 45 years (SD=11.78) ID: Mild-moderate-severe | 5 phases of improv training: -warm up -focusing -improvisation -reflection -ritual Group setting  Each session lasted 75 mins and took place every two weeks Drama exercises applied during first 3 phases, methods from family therapy used in the reflection phase | Participants were divided into two therapy groups in accordance to severity of intellectual disability and clinical estimation of emotional development: Group1: Participants required substantial support Group2: Participants required support. Simplified language Attention to physical reaction to questions Individual therapy sessions held at beginning of therapeutic process, and every 6 months, to reflect on personal process and therapeutic goals Ball games Mirroring  Synchronous movements Pantomime  Games with play identity Improvisation | N/A | Improv intervention found to be feasible and appropriate for adults with mild-moderate intellectual disability and mental health problems.  -High participation rate (19 months for group 1 and 20 months for group 2) | | | N/A | 4 | N/A | |
| 28. Carter (2022) | Dissertation. | Intellectual disability: defined as IQ<70 (unspecified)  Substance use problems. | Motivational interviewing (MI) Cognitive behavioural therapy (CBT) | Motivational Interviewing (MI): -Open ended questions, using simple, direct and concrete language. -Question topics should be specific, with limited number of consecutive questions allowing enough time for clients to respond. - Clinician flexibility and willingness to adapt to specific needs of client. - Clinicians should monitor clients for understanding and facilitate comprehension in a way that does not make the client feel incompetent. -Clear and concise language. -Verbal and non-verbal structural components. -Repetition of successful experiences to reinforce learning. -Facilitate discussions of change with examples and stories - clients may have difficulty imagining abstract future scenarios. - Being honest, genuine and empathetic. - Use of demonstrations - Use of role-play - Use of Visual aids  CBT: - Using simple phrases. - Additional psychoeducation. - Repetition. - Use of examples to explain concepts. - Involvement of caregivers. - Breaking down information into small chunks. - Providing individuals with choices (flexible approach to treatment). - Use of Simplified language. - Use of Visual aids. - Allowing the client ample time to process information.  - Increased number and length of sessions. Apply educational approaches that aim to prevent  substance abuse. | Adjustments should be made to support: - Verbal and non-verbal reasoning - Emotional understanding - Memory | N/A | | | N/A | N/A | N/A | |
| 29. Power et al. (2022) | Journal article.  Pre-and-post cohort study. Mixed methods. | N=25 Mean age: 33.3 years Intellectual disability: Borderline-mild-moderate  Participants had difficulty regulating emotion, difficulty oping with feelings, lack of social connection with others | Coping well group (CWD) included 6 weekly sessions that lasted 60-90 mins.  Provided service users with psychoeducation on emotions and mindfulness skills. Setting: Vocational centre | Maximum of one facilitator to three group participants - helped to aid in participants’ understanding of course materials. - Recap of sessions - cohort had to separate into two allowing facilitators to recap what had been discussed by adapting information to individual clients (easier in smaller groups). Minimum of two facilitators present for each session. Participants had option to include carers in group sessions - however, carers were asked d to support the service user to engage rather than engage with the material/content themselves.  Psychoeducation on emotions included.  Participants were encouraged to practice skills between sessions as homework. More resources for practical mindfulness exercises (e.g. printing pictures to use visual metaphors when explaining mindfulness). Creating facilitator manual to enhance standardisation of delivery. Easy-read booklets created for each session rather than distributing individual exercise handouts. | N/A | Quality of life (EUROHIS-QoL-8) score was significantly higher (t = 2.14, df = 21, p = 0.022) at post intervention (mean = 22.18, SD = 5.18) compared to pre-intervention (mean = 20.73, SD = 4.68). - Improvement in QoL. | | | N/A | 5 | N/A | |
| 30. Didden et al. (2022) | Book chapter | N/A | CBT | Attention:  -Shorter, more frequent sessions. -Reduce task length (smaller units). -Prevent distractions. Working memory:  -Use memory aids (e.g. visual prompts). -Present one task at a time. -Use short, simple, subject-verb-object sentences. Executive functions: -Used structured sessions (e.g. visual schedule). -Minimise switching between tasks, and redirect uninhabited responses.  Recognise emotions by using photographs of people's faces. Discriminate between thoughts, emotions, and behaviour by using personally relevant scenarios and pictorial stimuli. Make a connection between events and emotions, and recognise the mediating role of thoughts in the relationship between thoughts, emotions, and behaviour by using examples of situations, emotions, and behaviours that are congruent and by using pictorial stimuli. Include carers. Give homework. -Reduce amount of complexity in homework. Extend time length of sessions. Simplify psychoeducation. Trauma-focused CBT: Combined behavioural and cognitive techniques and focus on relationship between events and behaviour, cognitions and emotions. | N/A | N/A | | | N/A | N/A | N/A | |
| 31. Deb et al. (2022) | Book (descriptive) | Intellectual disabilities (unspecified) Dementia (pre-diagnostic stage - late stage advanced dementia) | N/A | N/A | Inclusion of carers (family, paid staff) Visual aids Role-play Psychoeducation Music therapy aromatherapy sensory stimulation Touch Use of electronic devices Domotica (smart homes) Entertainment Health discussions Rigid structured approach to care | N/A | | | N/A | N/A | N/A | |
| 32. Craven and Shelton (2020) | Journal article.  Single arm. Quantitative. | N=5 Mean age: 33.8 years Intellectual disability: Mild-moderate (IQ between 55 and 83) | Adapted DBT informed group-based skills programme Four modules: -Mindfulness -Managing feelings -Coping in crisis -People skills Manual contains 50 sessions delivered weekly. Mindfulness module was 12 sessions in length. | Simplified mindfulness techniques.  Repetition of mindfulness exercises - to moderate any cognitive or emotional arousal and enhance group engagement. Structured approach. | N/A | Improvements in emotional, psychological and behavioural domains, but not statistically significant | | | | 4 | N/A | |
| 33. Hackett (2012) | Doctoral thesis. Mixed-methods. Case-study. | N=4 Age: 21-28 years Intellectual disability: Mild  Includes measures of depression and anxiety. | 20 sessions of art psychotherapy | Simplified materials (shortened sentences, Shorter versions of assessment tools) Visual methods: - Participants produced drawings, both in colour and black and white, as well as clay sculptures to express emotions and convey thoughts. | N/A | Intervention did not improve symptoms of anxiety and depression consistently across cases. | | | 3 month follow-up showed improved relationships, improved personal motivation and positive outlook. | | | 8 |
| 34. Rossiter et al. (1998) | Journal article.  Single-arm.  Qualitative. | N=6 Age= 27-64 years Intellectual disability: mild-moderate-severe Anger | Simplified CBT approach for anger management. Eight weekly sessions lasting 1.5hrs.  Setting: Vocational centre | Visual methods (Diary included drawings, recordings of role-plays to play back and review body language and facial expression). Homework included practicing techniques from cassette tapes, and completing mood monitoring charts. Simplified language and concepts (use of metaphors, such as 'traffic light' to signal level of anger). Role-plays (for emotions and anger management). Breaks from therapy (2 week gap between sessions 6 and 7, 3 week gap between sessions 7 and 8). | More sessions. Ensure support staff attend all sessions. More role-play. Wider availability of anger management groups to more clients and staff. More varied timing (mornings, afternoons, evenings). Plan for more role-playing sessions. | Effective in reducing aggression as well as enjoyable | | | Two participants who received treatment in medium secure unit transferred to low secure unit within 20 weeks of completing treatment. 11-week follow-up showed maintained reductions in anger and aggression. | N/A | 8 | |
| 35. Willner et al. (2011) | RCT protocol. Two independent groups: EG: Manualised anger management group intervention CG: Support as usual | N=180 Intellectual disability: mild-moderate Age: unspecified  Number per group: 4-5 | 12 weekly psychoeducational group sessions for anger management with the inclusion of homework. Topics include: -Triggers that evoke anger. -Physiological and behavioural components of anger. -Behavioural and cognitive strategies to avoid the build up of anger and for coping with anger provoking situations. - Acceptable ways of displaying anger (assertiveness). | Visual methods - avoid writing wherever possible - Including pictures (PIS included stick people with no faces, as well as faces with facial expressions and no body) Presentations had heavy reliance on brainstorming (e.g. what makes us angry?) and Role-play. Simplified language. Homework assignments consisted of working with staff members to complete functional analysis ("hassle log"), of situation that angered them that week. - This was described, analysed and evaluated, using a pictorial work-book. | N/A | N/A | | | 6 month follow-up | | | N/A |
| 36. Gilrane-McGarry and Taggart (2007) | Journal article.  Single-arm. Qualitative.  Retrospective design. | N=11 Age: 25 - 72 years  Intellectual disability: Mild-moderate Bereavement | Semi-structured, one-to-one interviews with 11 people with intellectual disability. | Art therapy utilised for participants to express thoughts and emotions. Use of photographs in discussions about deceased loved ones. Family support. | Increase frequency of sessions. | Bereavement counselling offered by main-stream services found to be a positive and valuable experience.  - Support not routinely available for people with intellectual disabilities. | | | N/A | 5 | N/A | |
| 37. Moore et al. (1997) | Journal article.   Single-arm. Qualitative. | N=6 Mean age: 37.3 years Intellectual disability: Mild-moderate  Anger | 8 week anger management course, content included talking about feelings, understanding what makes people angry, how to cope with anger, practising anger management | Warm-up exercises (fun game at the beginning of the intervention for bonding between clients) Visual methods (Explaining concepts using pictures, including polaroids to show people angry and happy faces) Diaries for anger management  Role-plays by facilitators (Teach clients to understand when someone is angry, and what to do when people are angry) Practice of learned techniques Review of achievements | N/A | Reduction in number of anger-related incidences. | | | N/A | 5 | N/A | |
| 38. Cooper and Frearson (2017) | Journal article.  Case study.  Qualitative. | N=1 Age: mid 40s Intellectual disability: moderate  low-mood, comfort eating | 13 sessions lasting 1 hour of Compassion Focussed Therapy (CFT).  Two-phased: - Phase A: Assessments over 3 sessions, measures taken at 3 points in time. -Phase B: The intervention, including CFT formulation. Measures were taken 1-week post-intervention. | Visual materials (Colourful, visual diagrams) Repetition of verbal summaries. Frequent checks on understanding (Joe was asked to summarise frequently). Visual prompts given as homework ('to build a physical toolbox of strategies'). Reduced speed and content of sessions. Reduced depth and complexity of psychoeducation around CFT concepts. | Seek to meet the needs of the client, by seeking which components of therapy are deemed by the client to be most meaningful and effective. | Idiosyncratic measure (developed in conjunction with Joe, in pictorial form, to measure changes in mood) - Gradually increased from pre- to post-intervention, but remained relatively low.  Reduced comfort eating | | | 1 week follow-up, used to obtain feedback about the intervention. | 3 | N/A | |
| 39. Searle and Borseti (2021) | Journal article. Singe-arm. Quantitative. | N=9 Age: unspecified Intellectual disability: unspecified  Participants had difficulty regulating emotions, managing crisis, interpersonal effectiveness and had impulsive behaviours (e.g. self-harm and aggression)  Study included measures of anxiety and quality of life. | Weekly 2hr sessions of aDBT. Modules included: 1. Mindfulness skills in DBT 2. Please skills 3. Coping in crisis skills 4. Managing emotions skills Each module ran for 5/6 weeks. Average group size: 4.2 Total 16 sessions facilitated. | Group rather than individual sessions Taking into account individual needs Inclusion of breaks (15 min) | N/A | Significant reduction in psychological distress scores, but no significant difference in Anxiety, psychological well-being, or quality of life (WHO-QOL) scores pre to post treatment. | | | Increase in psychological well-being at 12 month follow-up | 3 | N/A | |
| 40. Lindsay et al. (2015) | Journal article. Two arms: EG:Trans-diagnostic treatment manual for people with intellectual disabilities CG: Treatment as usual, waiting list Quantitative. | EG:  N=12 Age=28.9 years ID: Mild (mean IQ= 62.4)   CG: N=11 Age= 33.1 years Intellectual disability: Mild (mean IQ= 63.9) Anxiety, depression | 8-14 sessions of trans-diagnostic programme for CBT with people with intellectual disabilities. | Inclusion of carers/family members. Role-play to rehearse strategies for difficult situations. Review of difficulties over previous week including homework assignments. Homework tasks made in collaboration with participants. Setting times for next session (flexible approach). | N/A | No significant difference in GSI values between groups.  Decrease in symptoms of anxiety and depression, although non significant (BSI), treatment group recorded higher values. | | | Study effects maintained at 3-6 month follow-up | 5 | N/A | |
| 41. Ali et al. (2021) | RCT. Two independent groups: EG: Befriending intervention  CG: Access to recourse booklet Quantitative. | N=16 Mean Age=  34.4 years (13.40% (CG)) 48.9 years (17.20% (EG)) Intellectual disability: Mild-moderate  Mental condition: -Depression: N=9 (56.25%) -Psychosis/Schizophrenia: N=3 (18.75%) -Bipolar affective disorder (N=1 (6.25%) -Anxiety disorder: N=5 (31.25%) -Autism: N=2 (12.5%) -ADHD: N=2 (12.5%) - Other: N=2 (12.5%) Country: UK | EGs matched with volunteer based on shared interests and availability.  Purpose: To provide emotional support and facilitate access to activities in the community. Pairs received recourse booklet of local activities and met once a week for 1 hour over a 6 month period, and spend at least half of the number of sessions in the community.  Minimum number of 10 meetings (average of 11.8). Semi-structured interviews also conducted for feedback on experience | Inclusion of 'befrienders' to support people with intellectual disabilities. | More support to be provided to volunteers through social events and opportunities to meet other volunteers that enhance peer support. Broader eligibility criteria for befriending. | All EGs:  -Reduced depressive symptoms (GDS-LD) -Improved self-esteem (adapted Rosenberg self-esteem scale -Improved quality of life (MANS-LD; WHOQOL-8) | | | 6 month follow up: - Effects maintained | 4 | N/A | |
| 42. Lewis and Rose (2018) | Journal article. Case study. Qualitative. | N=1 Age: 45 years Intellectual disability: Mild Anxiety | 10 sessions of Narrative therapy, unspecified duration of sessions therapy included 4 phases: 1. Defining the problem 2. Mapping the effects of the problem 3. Evaluating the effects of the problem's activities 4. Justifying the evaluation | Simplified language Regular checking of understanding Pacing the sessions and being flexible with session length | More sessions may be required for more severe intellectual disability. Slower paced sessions. | Decreased symptoms of anxiety post-session | | | N/A | N/A | 7 | |
| 43. Giannaki and Hewitt (2021) | Journal article CBT for individuals with intellectual disabilities and anxiety  Mixed-methods | N=4 Age: 21-56 Intellectual disability: Mild Anxiety | 7 week CBT sessions targeting anxiety. Group size: 4 | Inclusion of carers (parents, partner and paid support worker) Visual aids and prompts Diary sheets Shorter sessions Slower pace Concrete examples used Information repeated | N/A | Decrease in symptoms of anxiety | | | effects maintained at one-month follow-up | 5 | N/A | |
| 44. Fernandez et al. (2005) | Journal article. Case report.  Quantitative. | N=1 Age: 17 years Intellectual disability: Mild Major depressive disorder (MDD) | Weekly sessions of psychotherapy | Relaxation exercises during therapy  Multidisciplinary approach (Pharmacological and psychological treatment, including behavioural treatment) | N/A | Increased positive behaviours.  Improvement in all areas after 8 months of intensive treatment. GAF rose from 25 to 40. | | | 1 year follow-up - Participant stepped down from highest level of residential care from level 4-I (strictly supervised facility) to level 3 (less supervision). - Participant became more assertive, requesting change of residence. 2 year follow-up - Participant attended school 3x a week with reasonable success. - reported feeling bored and having difficulty being around problematic students - shows internalisation of controls and ability to consider consequences before acting.  - Participant engaged in minor self-harm behaviours when stressed or feeling abandoned. - No evidence or report of psychotic symptoms, and functioned fairly well with less medication than previously. - Fewer flashbacks related to sexual abuse and tolerated flashbacks better than previously. | N/A | N/A | |
| 45. Karatzias et al (2019) | Journal article. RCT (feasibility trial) Two groups: EG: EMDR plus standard care (SC)  CG: SC alone Mixed-methods. | N=29 EG: EMDR +SC (N=15)  CG: SC (N=14) Mean age: 42 years Intellectual disability: Mild-moderate PTSD | 8 sessions of EMDR lasting 1hr each | Bilateral stimulation - light bar, tactile and auditory stimuli | Make a range of information options available (e.g. symbolised information, easy-read materials, audio and visual options) Video explaining delivery of EMDR, indicating a range of options for bilateral stimulation (e.g. alternating flash lights, gentle tapping and sensory hearing bilateral stimulation. Discussion with families and support workers at an early stage should be initiated within the recruitment process (to address issues such as transport, in order to allow full participation In therapy) | EMDR found useful for people with ID and traumatic stress, particularly for symptoms of anxiety.  EMDR + SC found to be acceptable for use with people with intellectual disabilities. | | | 1 week post-treatment - 3 month follow-up, higher number of participants in EMDR+SC group diagnosis free compared to SC alone. | 4 | N/A | |
| 46. Jones and Finch (2020) | Journal article.  Pre/post-test within subjects design. Mixed-methods. | N=9 Age: unspecified Intellectual disability: Mild Anxiety, anger | 1hr sessions over 8-week period with a 2 week break in-between  Intervention incorporated elements from "I can feel good! skills training for working with individuals with intellectual disabilities" and adapted mindfulness techniques for people with intellectual disabilities  Setting: Vocational setting, including gym when necessary Group size: 9 | Simplified language (Easy-read materials) Structured sessions (consistency- Participants were aware of what to expect in each session, alleviating anxiety) Check-ins offered to participants at the beginning of each session (to discuss their week and any stressful situations and pleasurable activities) Homework (For participants to keep track of progress) | Smaller group size Consideration of environmental preferences for individuals.  Use of a gym for floor ant mat work when necessary.  Continuity of staff support. | Decreased anxiety symptoms for 6 participants (GAS-ID), two participants showed increased anxiety (May be due to personal circumstances for one participant) Increased mindful qualities for 5 participants, decreased mindful qualities for 2 participants | | | N/A | 4 | N/A | |
| 47. Jahoda et al. (2015) | Journal article  Behavioural activation for depressive symptoms in adults with intellectual disabilities (feasibility study)  Pre-post.  Quantitative. | N=21 Mean age: 42.2 years  Intellectual disability: Severe-mild-moderate Depressive symptoms | Weekly/fortnightly sessions held in the homes of clients. 10-12 sessions, main elements of therapy included self-monitoring through recording daily activity and mood, goal setting and activity scheduling. | Therapists worked with clients alongside a significant other. Intervention held in the homes of clients. Inclusion of carers (for at least 6 months) | N/A | Reduced symptoms of depression  Flexible number of sessions (either 10 or 12) | | | 3-month follow-up intervention outcomes maintained. One person exhibited increased depressive symptoms to baseline level. | 5 | N/A | |
| 48. Prangnell and Green (2008) | Journal article.  Case study. Qualitative. | N=1 Age: 54 years Intellectual disability: Mild Anxiety | 2 sessions of CBT per week. Attempted to gradually expose participant to source of anxiety (Dentist). Included cognitive restructuring, progressive muscle relaxation and In vivo exposure. | Hierarchy of exposure to dental treatment developed (Broken down into "several sessions" oriented towards increasing session duration). Collaboration with dental care team. Hierarchy printed into small booklet form and given to participant to take with him to each session. Shortened session duration of initial sessions (to ensure participant achieved the goals of these sessions). Longer sessions as participant progressed through treatment. Repetition of some sessions to consolidate achievements. Visual methods (flash cards). Role play and imagery (During assessment). Regular relaxation exercises. Use of "Stop signal" when particpant needed a break. | N/A | Reduced anxiety, Increased confidence. | | | N/A | N/A | 6 | |
| 49. Willner et al (2013) | Cluster RCT. Mixed-methods. | N=212 Mean age: 38 years Intellectual disability: Mild (FSIQ 57) | 12 weekly psychoeducational group sessions supplemented by homework.  Topics included triggers that evoked anger, physiological and behavioural components of anger, behavioural and cognitive strategies to avoid the build-up of anger and for coping with anger-provoking situations, acceptable ways of displaying anger. | Took into account individual needs and adapted content and flow of discussions to suit these - flexible therapeutic approach. Relaxation exercises (breathing exercises, counting to 10). Role-play. Multidisciplinary approach. | Shorter programme (fewer sessions). Include teaching materials. For more severe intellectual disability: further adaptation of communication - simplify language. | No significant impact on mental health, self-esteem or quality of life. Significant impact on anger coping skills - decrease in challenging behaviour. | | | 6-month follow-up: effects maintained. | 5 | N/A | |
| 50. Dillon et al. (2018) | Journal article. Semi-structured interviews. Qualitative | N=15 Age: 23-45 years intellectual disability: Mild-moderate Anxiety, depression | Group mindfulness Ran by two facilitators  Three mindfulness groups ran in different locations on an annual basis. | Group setting  Face-to-face Booklet containing meditations and 'thank you diary' - used to aid memory Visual aids Clear concrete language used in meditation instructions  Soul of feet meditation also used Not manualised, each session followed the same format to allow for rehearsal and repetition. | Involvement of caregivers - to aid in consistent practice of mindfulness techniques after sessions | Participants found it relaxing, enjoyed working in a group setting, but some found it upsetting to listen to other people's personal disclosures, and some didn't like how others would behave within the group. | | | N/A | 5 | N/A | |
| 51. Ho et al (2020) | RCT.  Two arms: EG: Expressive arts-based Intervention (EABI) CG: Waiting list  Mixed-methods | N=109 Mean age: 39.9 years Intellectual disability: Mild EG:  N=55 CG: N=54 Intellectual disability: Mild-moderate Country: China Low-mood, aberrant behaviour | EG group: 10 weekly 90-min sessions of arts-based intervention, 15 total contact hours - aimed to facilitate emotional awareness and expression through art Group size: 6-8 CG: Routine healthcare and rehabilitation services | Face-to-face modality  Visual methods: Drawings - participants showed low probabilities of using colours (86%) except for subgroup (14%) Two subgroups: 1. Pale subgroup (60%): 2. Colourful subgroup (40%) - both subgroups showed similar likelihood to use orange, yellow, green and brown colours. - Colourful subgroup more likely to use pink, blue and purple colours but less likely to use black colour than pale subgroup. - Colourful subgroup showed higher intensity of using colours such as pink, red, green and purple than pale subgroup. Participants in EABI group more likely to belong to colourful subgroup than to pale subgroup (not statistically significant). Rhythm or dance movements  Music games Create/revise art work or music pieces to represent thoughts and feelings. Structured approach (Structure kept similar). Content adjusted to suit individual needs. | N/A | No intervention effects on 8 mood states except anger. EABI group applied more colours in post-intervention drawings - similar pattern to health control groups in previous studies - Could possibly be due to enhanced emotional wellness of participants post-intervention. | | | N/A | 4 | N/A | |
| 52. McMahon (2021) | Doctoral thesis.  Mixed-methods. Three arms: EG1: Muse condition state mindfulness EG2: Headspace condition state mindfulness CG: No technology condition state mindfulness | N=4 Age: 19-21 years Intellectual disability: Moderate (unspecified IQ)  Stress. | Mindfulness sessions carried out via Zoom. Researcher virtually met each participant prior to beginning of study to go over how to use the provided technology.  Mindfulness incorporated wearable technology and mobile device.  Each session lasted 5 minutes and guided by former Buddhist monk. Total of 15 sessions occurred in the intervention phase. | 1. Guided mindfulness practice on mobile device 2. Wearable device designed to provide neurofeedback cues of mindfulness  'The MUSE 2 model' - wearable EEG device that measures brainwave activity and is designed for neurofeedback cues to help individuals practice mindfulness. Headspace app - used to test effect of technology supported mindfulness strategies Introductory sessions to mindfulness practice that focuses on the breath was implemented. Online modality (via Zoom). | N/A | Some participants experienced higher state mindfulness facilitated by neurofeedback cues. No functional relation for higher state mindfulness scores in either MUSE or Headspace condition for one Participant. No observed effects or significance on promoting higher state mindfulness scores in both MUSE and Headspace conditions, compared to no technology condition for all participants Some participants showed reduced stress. | | | N/A | 4 | N/A | |
| 53. Porter (2022) | Journal article.  Single-arm. Mixed-methods. | N=14 Age: 29-61 years (M=47.5) Intellectual disability: Mild-moderate  Trauma, PTSD - including undiagnosed Country: UK | 6-50 sessions of EMDR | Flexible treatment approach - allowed for therapy to be tailored to individual needs. Easy-read cards to take home-consolidate learning of concepts. Progressive muscle relaxation in addition to imaginal techniques - was preferred method of grounding. Inclusion of staff teams/partners/families to support participants during therapy. Drawings used in place of verbal description of target images - images not typically sought, and this was left out of standard protocol. Pictorial representations of emotions used to aid descriptions. - speech and language therapy support used in 2 cases to support this aspect of work. -Using emotions as targets for desensitisation is more effective than images or cognitions. Bilateral stimulation: -Self-directed butterfly taps, hand buzzers or therapist knee tapping. -Dual attention was effective, using a combination of light bar and hand buzzers. Ego-state interventions necessary for 2 cases, adapted using simplified explanations and drawings. Grounding enhanced with auditory, visual or tactile aids (e.g. a bell, a shell, catching cushion, standing and walking and looking around the room). Connecting traumatic events to concrete physical sensations, rather than an image or verbal memory of event. | N/A | Improved quality of life and reduction in symptoms. | | | N/A | 5 | N/A | |
| 54. Verhagen et al (2023) | Journal article Non-concurrent multiple baseline design. | N=9 Age: 34-53 years Intellectual disability: Mild PTSD | Weekly sessions of EMDR | Simplified language - to suit the needs of individuals with less language proficiency.  Icons/pictures used while explaining or inquiring. Bilateral stimulation - occurred only using eye-movements (e.g. fingers of the psychologist). Phases (3-8) repeated each session. | N/A | Reduction in PTSD symptoms and lower levels of daily life impairment. 6/8 participants no longer met criteria for DSM-5 PTSD post-treatment | | | 3-month follow-up: Study effects maintained. 7/8 participants no longer met criteria for DSM-5 PTSD. | 3 | N/A | |
| 55. Perera et al (2022) | Journal article  Report. | ADHD in people with intellectual disabilities (unspecified severity) | Personalised approach involves: - Psychoeducation - Pharmacological treatment - Behavioural and psychological interventions | Personalised approach - collaborate with carers/support networks Multimodal approach to treatment: Psychoeducation, Pharmacological management, Behavioural and Psychological interventions | Tailor treatment to suit individual needs | N/A | | | N/A | N/A | N/A | |
| 56. Hewitt et al. (2023) | Journal article.  Explanatory sequential design. Mixed-methods. | N=4 Age: 32-43 years Intellectual disability: Mild Lack of self-compassion | 8 weekly sessions of CFT face-to-face Last session recapped presented materials Intervention included homework, breathing exercises. Setting: Vocational setting | Sessions followed similar structure Visual and audio methods used (pictures, verbal explanations) Adapted protocol to incorporate specific interests of participants - improving engagement  Repetition (Three circles theory repeated) Use of concrete examples to illustrate points Clients encouraged to practice new skills between sessions Experiential exercises Simplified language (easy-read information) | More time could be spent on some content covered to consolidate understanding  Include carers (family members and support staff) | Improved psychological well-being, increased self-compassion | | | 1 month follow up, study outcome effects maintained | 5 | N/A | |
| 57. Acton et al. (2023) | Mixed-methods randomised controlled quasi-experimental design. Two independent groups: EG: Multi-component anxiety management (N=30) CG: Single component-adapted psychological treatment (treatment as usual) Mixed-methods. Feasibility study. | N=60 Age= 18+ Intellectual disability: Mild or moderate Mental condition= Anxiety, scores above 7 (range 7-18) on the anxiety component of the Moss-PAS-ID | All EGs to receive 12 face to face sessions over a 10 week period using co-developed novel anxiety management manual for delivery of treatment sessions. Each session to began and ended with breathing exercises. After completion of intervention, participants will be invited to attend focus groups or individual interviews. Semi-structured interview questions will be used.30 participants per arm. | Breathing exercises after every session scenario cards Psychoeducation | N/A | N/A | | | 12 week follow up assessments | 3 | N/A | |
| 58. Everett et al. (2021) | Book chapter | Individuals with Intellectual disabilities (IQ<70, significant impairment of adaptive behaviour) | N/A | Simplified language Inclusion of carers/family members Visual methods (pictures) Formulation jigsaw to convey session plan using images  Accessible sources for psychoeducation - using visual resources (e.g. YouTube) Practical exercises - visual handouts, easy to follow step-by-step guidance on more mental based activities, easy to access and practice at home | N/A | N/A | | | N/A | N/A | N/A | |
| 59. Loeper and Schwartz (2023) | Journal article. Semi-structured interviews. Qualitative. | N=4 Age: 19-23 years Intellectual disability: unspecified Anxiety, depression, OCD | Peer-mentors with Intellectual disabilities helped develop a peer mentoring intervention for people with ID | Incorporating peer-mentors with intellectual disabilities into interventions for mental health | N/A | Mentors developed sense of pride, enjoyment and benefits made to professional development - potential positive mental health benefits | | | | 5 | N/A | |
| 60. McNeel et al. (2023) | Book chapter | Children | CBT | Multi-modal treatment (e.g. online, face-to-face). Frequent sessions. Use of concrete examples. Behavioural intervention plans. Positive behaviour supports (PBS). Simplified language. Slower paced sessions/lengthened treatment. Caregiver involvement. Visual strategies. More emphasis on behavioural components. Goals should focus on improving individual's quality of life instead of, or in a addition to adhering to social Normas. - Treatment goals should be socially and culturally valid. Teaching strategies (e.g. prompting and modelling). Teaching various intervention processes to develop skills necessary for treatment (e.g. simulated exposure therapy using neutral instead of feared stimulus). - Repetition may be necessary for learning trials to consolidate learning. Verbal, visual and physical prompts (level of prompting reflective of the assistance needed for client to complete skill) - Type of prompt used will be determined by what is being taught as well as child developmental level). - Verbal prompts used if participant is able to complete tasks independently.  - Visual prompts (e.g. gestures, pictures) - Working with children who do not understand verbal prompts and is unfamiliar with skill. - Physical prompts (e.g. hand-ver-hand guidance) - Help client practice movement required. Teaching and practicing social skills or social communication. Contingent reinforcement (providing rewards for engagement in specific tasks) - increase motivation. | N/A | N/A | | | N/A | N/A | N/A | |
| 61. Goad and Parker (2021) | Journal article.  Pilot study. Mixed-methods. | G1: N=3 G2: N=3 Age: 19-39 years  Intellectual disability: Mild Low-mood, Self-criticism, shame | 10 sessions of compassion-focused therapy  Setting: Community base | Increased number of sessions (from 6 to 10) Recap of sessions | Use person-specific goal attainment outcome measures | Increased self-confidence | | | N/A | 4 | N/A | |
| 62. Buijs et al. (2021) | Journal article. Case study.  Mixed-methods. | N=2 Age: 19 and 21 Intellectual disability: One participant=Mild One participant= Average intellectual functioning 22q11.2DS, ADHD, Anxiety, OCD, Phobia, Social anxiety disorder (SAD) | 13 weekly sessions lasting 45-60min of CBT, using guidelines for GAD and SAD. Participants expected to practice new skills between sessions (homework) | Child's version of ORS/SRS worksheets for people with intellectual disabilities Proactive approach Focus on activation Concrete use of language Involvement of parent Multidisciplinary approach (Physicians, psychologists, social workers) Additional time per session (for preparation) | Tailor needs to specific clinical characteristics and social context of individual Flexibility and proactive approach | Increased awareness of emotions, no changes in behaviour. Observations contradicted written assessments - suspected to be reflective of poor comprehension of written materials | | | N/A | N/A | 8 | |
| 63. Unwin et al. (2023) | Journal article. Feasibility study. Mixed-methods. | Carers Clients - unspecified intellectual disability Therapists Clinicians | Psychoeducation and emotional stabilisation-Eye movement desensitisation and reprocessing. (PES-EMDR)  10 weekly sessions for first phase (PES) and aspects of phase 2 and 3 (preparation and assessment), followed by 10 weekly sessions EMDR (flexibility to increase sessions if needed). | Blended approach (online and face-to-face). Inclusion of psychoeducation component and emotional stabilisation - purpose to install strengths and resources, stabilise emotional regulation and build alliance and trust. Flexible treatment approach - possibility to increase phases if needed (e.g. multiple traumas present in clients/other comorbidities present). Stages, language and outcomes made more accessible. Not favouring side-to-side finger movements over other forms of bilateral stimulation (e.g. tapping). Encouraging creative use of expression (e.g. techniques from art and narrative therapy/storytelling). Involvement of carers where appropriate to support clients within/between therapy sessions. | N/A | Decrease in PTSD symptoms and increase in psychological well-being and positive life changes. | | | N/A | 3 | N/A | |
| 64. Ali et al (2023) | Journal article.  Exploratory qualitative research. | N=3 Intellectual disability: unspecified severity | Four online meetings lasting 1hr, included training sessions and discussions about support for people with intellectual disabilities in the context of mental health | Easy read summaries after each meeting. Simplified explanations of content  Involvement of people with intellectual disabilities to support those with intellectual disabilities seeking treatment for mental heath.  'Ground rules' for talking about personal experiences, with emphasis on confidentiality - Limits potential 'emotional triggers', and allows people to be comfortable talking about their experiences. | More time to write about experiences More meetings in person rather than online | Increased confidence in people with intellectual disabilities. | | | N/A | 5 | N/A | |
| 65. Charlton & Dykstra (2011) | Single arm within subjects pre-post design  Mixed methods  EG: Dialectical behaviour therapy (DBT) | N=19 Age group: Children and adolescents Intellectual disability: unspecified level | 30-60 min sessions of DBT, increased sessions per week (two per week) | Auditory and visual information Simplified language Structured therapy sessions Directive and active approach Activities (modelling, role play) Reduced sessions (30-60 min), at higher frequency (2 times per week) Inclusion of carers Review of daily diary cards as part of individual therapy instead of group Environmental consistency | N/A | Greater insight into situations, thoughts, emotions and actions, reduction in symptoms of depression | | | Three Participants lost at follow up, | 3 | N/A | |
| 66. Essau and Longhi (2013) | Book chapter | Williams syndrome  Intellectual disability: Moderate | CBT | Simplified language. Visual illustrations (e.g. drawings to illustrate main principle of CBT) - Stories used to teach patients how to react to fearful situations. Include homework assignments and in-session activities that do not involve high level of physical activities. Structured description of each session and according to participants' individual needs. Repeat direct instructions and reminders. Include two sessions on social skills training. Remind individuals of next session (e.g. number of night sleeps). | N/A | N/A | | | N/A | N/A | N/A | |
| 67. Kirk et al (2014) | Single-arm within subjects pre-post design. EG: Anxiety management group  Quantitative | N=5 Age: 20-53 years Intellectual disability: Mild-moderate Anxiety Country: UK | EG: 8 sessions lasting 2 hrs, included 20 min breaks Included emotional awareness, cognitive mediation, increase self-awareness | Slower paced Longer breaks Smaller group size Well defined beginning, middle, and end Simplified concepts and language Easy-to-understand materials Simplified therapeutic procedures  Working in a concrete and multi-sensory way (e.g. pictures, board games, art) Applying skills to real life situations Accessible homework tasks Three facilitators Recap sessions Inclusion of carers | N/A | No significant reduction in anxiety levels (GAS-ID). Increased mean GAS-ID score | | | 2-month follow-up Effects maintained, evidence of increased knowledge about anxiety and coping strategies | 3 | N/A | |
| 68. Dilly (2014) | Case study. Quantitative. EMDR. | N=1 Age: 25 Intellectual disabilities: Unspecified, however general cognitive ability, verbal comprehension, attention and mental control noted to be 'extremely low range'. Trauma, PTSD, self-harm Country: UK | 12 sessions lasting 40 mins EMDR Setting: Vocational clinic | Safe place visualisation technique' developed to manage arousal - laminated picture of "woods" used in conjunction with scented fir cone to aid visualisation. | N/A | Reduction in symptoms of PTSD | | | No-follow up | 5 | N/A | |
| 69. Barrowcliff & Evans (2015) | Case study.  Quantitative. | N=1 Age: In her 40s Intellectual disability: Moderate-severe Trauma, PTSD Country: UK | 8 phases of EMDR | Inclusion of carer  More preparation time (Couple of sessions) Several weeks of preparation, in order to practice techniques used in therapy (e.g. distancing, safe place identification) Assessments during therapy application to evaluate change and understand treatment efficacy Different methods of bilateral stimulation (alternating hand-taps) Observation of discernible levels of distress | N/A | Decreased symptoms of PTSD | | | No follow up | 5 | N/A | |
| 70. Buhler (2014) | Doctoral thesis.  Multiple-baseline, ABA, experimental design.  Quantitative | N=6 Age: 18-65 years Intellectual disability: Mild-moderate PTSD | 8 Phases of EMDR. Each session lasted 28-60 min Setting: Vocational setting | Technology: - Basic motion logger wrist watch (Features included an event marker, audible feedback) - Motionlogger interface (Device that connected to computer via USB cable and works with associated software) - Blood pressure monitor - Sensors - Video recording equipment (recording of sessions) - EMDR bilateral stimulation equipment (Induced visual, auditory or tactile BLS) Flexible therapeutic approach. - progression through EMDR phases was based on client stability (taking into account individual needs) Inclusion of carers. Repetition | Allow 90 min sessions and accumulating information on best practices in adapting EMDR protocol for people with intellectual disabilities. | All 6 cases showed improvement. - All lost diagnoses of PTSD and all improved along a number of dimensions of psychopathology. | | | 6 week follow up-intervention effects maintained. | 5 | N/A | |
| 71. Lew et al. (2006) | Single-arm cohort study. Quantitative. | N= 8  Age: 25-61 years Intellectual disability: Mild-moderate Depression, schizoaffective disorder, personality disorder Country: USA | Purpose:  1. Teaching and strengthening new skills 2. Addressing motivational and behavioural performance  Individual therapy: 1 hr weekly sessions - flexible, meeting twice a week 30 min sessions Setting: Work, or home  Group therapy: 23 weekly 2 hr sessions, inclusion of co-leaders, staff and parents Included Mindfulness, distress tolerance, emotion regulation, interpersonal effectiveness, celebration. | Simplification of language (e.g. simplified diary card) Involvement of parents/carers Breaks included  Repetition for skills acquisition  Visual and audio methods (e.g. film, music, pictures) | N/A | Increased risky behaviours in first 6 months, improvements after 12 months. | | | N/A | 5 | N/A | |
| 72. El-Tahir and Bayley (2017) | Case study. Qualitative. | N=1 Age: 43 years Intellectual disability: Mild Trauma, emotional dysregulation, BPD | Weekly sessions for 18 months Therapy aimed to 'separate the person from the problem', and alter patient's interpretation of traumatic events that occurred, and symptoms. | Visual material Drawing Role play Shorter sentences Simpler sentences Repetition | N/A | Improved anxiety, mood changes, sleep, eating and drinking | | | Engaged with relevant health care professionals for 4.5 years, no further inpatient treatment required, reduced frequency of GP and A&E services, decrease in affective or neurotic disorder | 5 | N/A | |
| 73. Florez and Bethay (2017) | Case study. Mixed-methods. | N=1 Age: 28 years Intellectual disability: Mild-moderate Anxiety (GAD), intermittent explosive disorder (IED) Country: USA | 1hr weekly DBT over 12 months first month: assessments, agreements, building rapport months 2-3: Mindfullness 3 Months: emotional regulation skills 1 month therapy break Mindfulness post break, followed by interpersonal effectiveness module during next 3 months Last 2 months: distress tolerance module | Visual and concrete prompts - associated with escalation of emotion Systematic steps on how to identify emotional states Repetition Practical activities Constant consolidation of understood concepts Simplified language Experiential exercises (e.g. mindful walking, body scan) | Multidisciplinary approach recommended | Improvement of mood, better coping of problems and decrease in challenging behaviour | | | Referral to next graduate intern to continue DBT treatment. Average of less than one challenging behaviour maintained after 1-year of treatment | 5 | N/A | |
| 74. Hewitt et al. (2019) | Single-arm  EG: Adapted Dialectical Behaviour Therapy (DBT) Mixed-methods. | N=4 Age: 24-48 years Intellectual disability: Mild Emotionally unstable personality disorder, OCD Country: UK | EG: Adapted Dialectical Behaviour Therapy (DBT) , 18 sessions over 6 months comprising 4 modules: 1. Mindfulness 2. People skills 3. Managing feelings 4. Distress tolerance Interviews also held with five identified themes: 1. Remembering vs forgetting 2. Thinking about the future 3. Group as a positive or negative experience 4. Personalisation and adaptation 5. Continuity and impact of group | Inclusion of carers. | N/A | Decreased distress scores | | | Intervention effects remained low compared to pre treatment at 6 month follow-up, however increased after 2 year follow-up | 5 | N/A | |
| 75. Panditaratne et al. (2022) | Single-arm  EG: CBT ("Fearless Me!") Qualitative. | N=8 Age: 8-17 years Intellectual disability: Borderline-mild-moderate Anxiety Country: Australia | 10 sessions of CBT, including 10 face-to-face sessions and an online simplified-CBT program. Included teaching material, homework, relaxation techniques, identifying and challenging unhelpful/irrational thoughts, developing and implementing exposure hierarchies. Interviews held for further information about intervention experience. Setting: Vocational centre | Breaking instructions into smaller steps (simplified CBT process) Vital cues Involvement of carers (parents) Multiple modalities (face-to-face, online) Exercises conducted on website (homework) Simplified language | N/A | Positive experience of intervention overall, reduction, some showed decrease In anxiety and improved emotion regulation, some showed no changes. | | | No follow-up | 5 | N/A | |
| 76. Unwin et al. (2023) | RCT - Protocol evaluation EG: EMDR Qualitative. | N=5 Intellectual disability: Mild-moderate | 10 weekly sessions of Psycho-education and stabilisation (PES), includes EMDR. | Inclusion of carers Visual supports Frequent "return to target" Focus on emotions and physical sensations rather than mental images or cognitions Simplified language Tapping (bilateral stimulation) Multi-modal (Face-to-face and online) | N/A | PES received well overall. Some difficulty in experience of reprocessing (clients reported feeling "overwhelmed". Decrease in PTSD symptoms reported | | | N/A | 5 | N/A | |
| 77. Newsome Hoyle and McKinney (2015) | Single-arm. Music therapy.  Quantitative | N=3 Age: 33-60 years Intellectual disability: moderate-profound Bereavement  Country: USA | 9 sessions of Music therapy Group size: 3 | Visual and audio methods (picture book, music, icons) Language simplification (direct, simple questions) | N/A | Decrease in negative behaviours (BPRS-DD) in one participant, two showed no decrease in negative behaviours, but improved social skills | | | 4-week follow-up treatment outcomes maintained | | | |
| 78. Hagiliassis et al (2005) | RCT Two independent groups: EG: Anger management program ("The anger management training package") CG: Waiting-list, treatment as usual Quantitative | N=29 Mean age: 44.93 years Intellectual disability: Mild-moderate-severe Country: Australia | EG: Anger management program, 12 sessions, 2hr duration with 15 min breaks. | Visual methods - pictographic symbols (visual learning aid) Active learning techniques - role play, repetition Involvement of carers | N/A | EG: Improved levels of anger CG: No significant difference in anger levels | | | Effects maintained after 4-month follow up | 4 | N/A | |
| 79. Boulton et al. (2018) | Single arm - feasibility study  Pre-post design.  Mixed-methods. EG: Acceptance and commitment therapy (ACT)- photography based | N=6 Mean age: 32.33 years Intellectual disability: unspecified, however all had IQ<70 psychosis/mania, suicidality or serious self-harm behaviour Country: UK | EGs:  -6, 30min sessions of manualised values-based intervention on one-to-one basis.  -Took photos of valued aspects of life -Met with therapists and undertook "catching what matters" Setting: Either vocational centre or home | Visual methods - Photography, pictures | N/A | Participants with intellectual disabilities were able to engaged with intervention. Understood abstract concept of values. Implemented self-directed photography skills to capture valued aspects of life. Enhanced therapeutic engagement. | | | No follow up | 5 | N/A | |
| 80. Weeland et al. (2015) | RCT (protocol) 'Op Volle Kracht' (OVK)  Two groups: EG: OVK + treatment as usual CG: Treatment as usual | Both groups: N=91 (Each group), 182 total Age: 12-16 years Intellectual disability: Mild-moderate (IQ 50+) Depression, anxiety Country: Netherlands | OVK (based on CBT). 8 sessions lasting 45 min. Aims to alter maladaptive thoughts, feelings and behaviours by teaching skills to recognise automatic negative thoughts and the consequences on feelings and behaviour. 10 groups Setting: Vocational centre | Shortened treatment duration (from 16 to 8 sessions) Simplified materials Inclusion of carers in homework exercises Personal implementation plan More attention on personal experiences of youth Visual methods (pictograms, video clips) Group talks Individual and group exercises Simplified language Skills taught in smaller sub-steps  Techniques requiring higher level of cognitive reasoning (e.g. reasoning about hypothetical situations) are replaced by concrete and behaviour-oriented techniques (e.g. teaching self-instruction and behavioural alternatives in situations) | N/A | N/A | | | 3 month follow-up | 3 | N/A | |
| 81. Vereenooghe and Westermann (2019) | Journal article.  Single group pre-post design. Feasibility study. Mixed-methods. EG: Digital mental well-being intervention. | N=15 Median range= 19 years Intellectual disability: Mild-moderate Unspecified mental illness  - Intervention described to improve psychological well-being | 1 session of interactive mental well-being intervention per week, maximum of 2 per week for 4 weeks. First session started with interactive introduction to learn how to use the tablet, followed by module 1 (Participation- understanding that participation may lead to improved social contact and social support as well as being enjoyable). Module 2: Being active. - Aim: Improving mental well-being through increasing physical activity. Module 3: Friendships.  - Aim: Highlighting the importance of a supportive network and tips for maintaining friendships. Module 4: Relaxation. - Aim: To acknowledge the need for relaxation and how this can be achieved. Module 5: Self-acceptance. - Aim: Addressing and accepting your strengths and weaknesses, including your disabilities. Module 6: Communication. - Aim: Being able to communicate your needs using a positive communication style. Module 7: Self-actualization. - Aim: Behavioral activation and finding of activities that support your personal development. Module 8: Cognitive restructuring. - Aim: Identifying and changing helpful and unhelpful thoughts to help change how we may feel about a situation. | Delivered online  Sessions are brief  Choice of activities, so that those with intellectual disabilities can choose tasks relevant to them (flexible approach)Inclusion of support workers (carers) - provided practical and content support (e.g. clarifying content, help use tablet computers, prompting user to select on-screen response etc.). Website offers compromise between rigid and flexible approach - presenting options for local activities, giving participants the option to choose those tasks that are more relevant to them. - Interactive content - Audio-visual methods - psychoeducation conveyed through avatars on website | More sessions for longer intervention period | Website feasible for use with people with mild-moderate intellectual disabilities. Improvements in subjective well-being and reduced behavioural problems observed in some participants | | | No follow up | 4 | N/A | |
| 82. Verberg et al. (2018) | RCT (protocol) Two groups: EG: Online mindset intervention ("Growth factory") CG: Care as usual | N= 60 (for each group) Age: 12-23 years Intellectual disability: mild-borderline (IQ 50-85) | 6 sessions of online "The growth factory", each session lasting 25-40 min.  Improve confidence and self-esteem - education about plasticity of brain and ability to change   Setting: Vocational centre | Modality - online Visual methods (game-like, inclusion of avatars to teach concepts) | N/A | N/A | | | 3-month follow-up 6-month follow-up | 3 | N/A | |
| 83. Roberts and Kwan (2018) | Single-arm. EG: Adapted CBT. Quantitative. | N=13 Mean age: 27.61 years Intellectual disability: Mild-moderate Anxiety Country: Australia | 6 week CBT-ID group programme lasting 1.5 hrs each. Group size-2-4 | Visual methods (icons, pictures) Simplified language (use of metaphors) Activities, worksheets, role play and homework tasks (used to identify and link thoughts, feelings and behaviours in response to anxiety-provoking situations) | N/A | Decreased symptoms of anxiety | | | No follow-up included | 4 | N/A | |
| 84. Mevissen et al (2012) | Single-arm EG: Eye movement desensitisation and reprocessing (EMDR) Qualitative. | N= 4 Age= 10-49 years Intellectual disability: Severe Trauma, PTSD | All participants: Weekly 60 min sessions of EMDR, number based on clients needs (3-14). Carers included, goal of treatment to resolve symptoms from disturbing and unprocessed life experiences. | Simplified language (adapted instructions to cognitive and emotional level of clients) | N/A | Decrease in PTSD symptoms | | | Follow-up 6 weeks - 2 years, treatment effects maintained, some improved further. One participant had increase in aggressive outbursts at 16 months. | 5 | N/A | |
| 85. Idusohan-Moizer et al (2015) | Two groups: EG1: Mindfulness-based cognitive therapy (MBCT) EG2: Mindfulness-based cognitive therapy Mixed-methods Non-randomised | N=10 Age= 21-44 years Intellectual disability: Borderline-mild-moderate Depression, anxiety Country: UK | Group therapy MBCT, 30 min sessions for 9 weeks. program aimed to improve mental well-being, reduce anxiety and depression | Easy-to-read materials Visual methods (Pictures) Exercises on developing compassion Meditation on the soles of the feet | Regular feedback from participants on clarity of sessions and caterials. More repetition. More role-play. Facilitators would need to cater separately for people with different levels of ability. | Significant decreases in anxiety.  Decreases in symptoms of depression. | | | 6-week follow-up sustained intervention outcomes | 3 | N/A | |
| 86. Hronis et al (2019) | Case series design. Two independent groups: EG1: Junior group - CBT EG1: Senior group - CBT Quantitative. | N=21 Age= 12-18 years Intellectual disability: mild-moderate Anxiety Country: Australia | Both groups: CBT-based program adapted for children and adolescents with intellectual disabilities ("Fearless me! program"). 10 face-to-face 45 min sessions over 6 weeks. Involved teaching materials, group or individual activities, use of online program. | Multiple modalities (face-to-face and online) Frequent sessions (twice a week) Material presented verbally and visually Role play and hands on activities Use of videos  Concrete examples of cognitive restructuring  Simplification of concepts (broken down into component parts) Repetition of tasks Text-to-speech function on website | N/A | Significant reductions in anxiety | | | No follow-up | 3 | N/A | |
| 87. Hassiotis et al (2011) | RCT (Protocol) Two independent groups: EG: CBT CG: Treatment as usual | EG: N=15 CG: N=15 Age: 18+ years Intellectual disability: Moderate - severe Depression, anxiety Country: UK | EG: 16 weekly one-to-one 60 min CBT sessions over 4 months. - 15 min interviews with open-ended questions for feedback. | Visual methods Role play Simpler questioning styles Slower-paced therapy | N/A | N/A | | | 2 month follow up | 3 | N/A | |
| 88. Hartley et al (2015) | RCT Two independent groups: EG: Group CBT with caregiver component CG: Treatment as usual | N=24 Mean age: 44 years Intellectual disability: Mild (IQ= 50-73) Depression, anxiety Country: USA | CBT intervention ("Empower") 10 weekly meetings lasting 1.5hrs.  Caregivers integrated within treatment: 1) Learned about CBT and rationale behind activities 2) Supported adults with ID in CBT group 3) Given instructions (homework, in-between sessions) about how to support adults with intellectual disabilities. | Simplified language Checking understanding Use of real-life examples Visual materials | N/A | EG: Significant decrease in depressive symptoms CG: No significant changes in depressive symptoms | | | 3-month follow-up: Treatment outcomes maintained | 4 | N/A | |
| 89. Cooney et al (2018) | Single-arm. Computer-assisted CBT (cCBT). Qualitative. | N=24 Mean Age: 42 years Intellectual disability: Mild-moderate Anxiety, depression  Country: Ireland | cCBT, Participant comes across 6 different characters who teach 7 core CBT skills (e.g. differentiating between thoughts, feelings and behaviours) 7 1hr sessions. Setting: Vocational clinic | Audio and visual delivery methods (computer game) Simplified language and concepts Between therapy skills practice using workbook (written or pictorial sticker responses) | N/A | Increased awareness of thoughts, feelings and behaviour, reduction in depression symptoms | | | Changes maintained at 3-month follow-up | 5 | N/A | |
| 90. Clapton et al (2018) | single-arm non-randomised.  Compassion focused therapy (CFT) - two rounds of groups ran consecutively on one site due to small referral number in first round. Mixed-methods. | N=6 Mean age: 38.5 years Intellectual disability: Mild Anxiety, depression Country: UK | 90 min of CFT, three systems: 1. threat and self protection 2. drive seeking and reward  3. affiliative/soothing | Visual methods (e.g. pictorial psychoeducational material) Minimise use of abstract language (simplification) Supporting workbook (written ad visual summaries of sessions) | N/A | Decrease in self-criticism and unfavourable social comparisons | | | No follow-up | 5 | N/A | |
| 91. Carrigan & Allez (2017) | Case study. Quantitative. | N=1 Age: 26 years Intellectual disability: Mild PTSD, anger Country: UK | CBT, 12 weekly 1hr sessions. - initial session simplified model of PTSD - Shifted focus to consider anger outbursts - Psycho-education Setting: Vocational centre | Language simplification (metaphors) Psychoeducation Imaginal reliving of events | N/A | Improved symptoms of PTSD and aggression | | | No follow up | 5 | N/A | |
| 92. Brown and Hooper (2009) | Case study. Quantitative. | N=1 Age: 18 years Intellectual disability: Moderate-Severe Anxiety Country: UK | 17 session over 6-month period. Acceptance and commitment therapy (ACT) - regular mindfulness skills practiced | Exercise - Inclusion of walks during sessions High levels of prompts to focus on tasks Activity-based exercises Increased number of sessions (17 over 6 months) Experiential exercises | N/A | Reduced anxiety, shorter duration of rumination | | | 4-month follow-up, Intervention gains maintained | 5 | N/A | |
| 93. Blakeley-Smith et al. (2021) | Single-arm pre-post within subjects design. Quantitative. | N=23 Mean age: - Beginning of treatment: 15.92 years - Completion of treatment: 16.61 years Intellectual disability: Moderate Anxiety  Group size: 2-4 adolescents and parents with two facilitators | EGs participated in 14-week 40-60 min CBT group intervention. Intro to common CBT strategies for anxiety, focus on use and generalisation of psychoeducational strategies, graded exposure to improve anxiety management, stakeholder input (e.g. parent treatment acceptability rating). Group size: 2 to 4 Setting: Vocational centre | Shortened session length (45-60 mins) Reduced group size (2-4) Parents and teens participated together Parent only curriculum front loaded onto program (3 parent sessions preceded 11 sessions of parent-teen dyadic work, breakout parent/teen session took place at week 7) Somatic management Visual structure (pictures, videos) Repetition | N/A | High completion rates, improved anxiety | | | No follow up | 5 | N/A | |
| 94. Baldwin et al. (2021) | Two-arm RCT Protocol EG: Healthy mind program CG: Waiting list, Treatment as usual | N= 167 Age: 16+ Intellectual disability: Borderline-mild Depression, anxiety | EG: Healthy mind program (online) 8 week period. Five interactive activities: 1. Recognising feelings 2. Breathe and relax 3. Taming anger 4. Having more fun 5. Tackling unhelpful thinking  CG: Waiting list, treatment as usual over 8 week period. | Online Simplified language Interactive activities Visual (pictures, videos, animations) and audio methods of delivery Inclusion of carers/supporters Psychoeducation | N/A | N/A | | | 3-month follow up | 4 | N/A | |
| 95. Mevissen et al (2020) | Single case study. EG: Trauma treatment (KINGS-ID) Quantitative. | Total: N=19  Parents: N=9 Age= 33-48 years  Children: N=10 Age=2-16 years  Both: Intellectual disability: Mild Trauma Setting: Vocational centre | 8 week treatment, starting with 6 week inpatient programme. Included EMDR for severe trauma, children assisted by family caregivers with background in CBT training. Safety, opportunities for choice, collaboration, empowerment, attention for trauma triggers, understanding emotional and behavioural problems. Two weeks parent support at home Setting: Vocational centre, at home | Inclusion of carers (2 week parenting support at home) Simplified language Visual cues Focus on one task at a time (systematic approach) | N/A | Significant decrease in trauma symptoms for both parents and treatment | | | Results maintained at 6-month follow-up | 5 | N/A | |
| 96. Mayer et al (2023) | RCT Two-groups EG: Narrative exposure therapy CG: Waiting list | EG:  N=7 Mean age: 46 years Intellectual disability: Unspecified CG: N=8 Mean age: 49.8 years Intellectual disability: unspecified Trauma - undiagnosed Country: Switzerland | EG:  1 hr 4-9 sessions (lifeline development) 9-27 sessions (working through traumatic events). | Simplification of language (shortened sentences, simple everyday words, using the same words consistently, avoiding technical terms) | N/A | Decease in PTSD symptoms No significant difference compared to control | | | No follow-up | 3 | N/A | |
| 97. Kneuer (2014), EASA | PowerPoint presentation. | N/A | N/A | Simplify language, speak clearly and slowly - allowing space within the conversation. Speak calmly and in a normal tone.  Avoid abstract ideas and Jargon. Be specific. Be aware of body language. Increase frequency of sessions - more time to establish a therapeutic relationship.  More sessions per week for brief periods of time recommended than one session weekly. Shorter sessions - may be harder to maintain ps attention and focus on therapeutic issues that last the usual 40-60 minutes. Allow flexibility in length of therapy sessions. - 30 minute sessions recommended for some people. Increase length of treatment:  - to allow for repetition. - to allow for newly acquired skill sets to be generalised. - to build upon therapeutic relationship and needed time to work on goals and objectives. - effective termination process may take longer. Utilize more structured and directive approach. Communicate with other care providors (e.g. care providers, parents, psychiatrist, residential staff etc). Modify complexity of information to suit the person's developmental framework. Use visual supports (e.g. graphics and pictures, flip charts, games, social stories, handouts, multisensory approach) | N/A | N/A | | | N/A | N/A | N/A | |
| 98. Hassiotis et al (2012) | Manual of CBT for people with intellectual disabilities and common mental disorders. | N/A | CBT | Be more didactic and present key concepts in extremely concrete ways. Provide extra support in the form of visual aids (e.g. pictures, drawings and signs for certain tasks such as mood monitoring, presenting temporal concepts, and identifying automatic negative thoughts). - Use images such as those in photosymbols collection and include many examples of these in worksheets. Take therapy at a slower pace, using repetition, and encouraging 'overlearning' in some scenarios. - aim to enhance client engagement and motivation in therapy. include psychoeducation and relaxation training. Simplify language - use simple, straightforward, everyday language and limit number of key concepts and ideas (no more than three per sentence). - Use concrete examples. - Contextualise information with examples as often as possible - helps clients rememeber what is being said even if the words aren't fully comprehended. Focus on non-verbal communication - tune in to body language of clients and other mechanisms of clients. | N/A | N/A | | | N/A | N/A | N/A | |
| 99. Willner et al (2002) | Journal article. RCT  Two arms: EG: Cognitive behavioural anger management group CG: waiting list  Quantitative Group size: 7 | N=14  EG: Mean age: 31.4 (SD=14.2) Intellectual disability: Mild (IQ= 63.9 (SD=8.9))  CG: Mean age: 30.4 (SD=12.4) Intellectual disability: Mild (IQ=65.3 (SD=9.3))  Anger | 9 weekly 2 hour sessions. All sessions began with a warm-up exercise and were punctuated by a tea/coffee break. At the end of the first session, participants were taught a relaxation exercise, and encouraged to use it at home; all subsequent sessions ended with the same exercise.Topics addressed over sessions included: aims and rules for the group; the triggers thatevoke anger; physiological and behavioural components of anger; behavioural and cognitive strategies to avoid the build-up of anger and for coping with anger-provoking situations; and acceptable ways of displaying anger (assertiveness). Presentation relied heavily on brainstorming (e.g. ‘What makes us angry?’) and, in later sessions, role-play. Sessions two to nine included some revision of material covered in the previous week. Towards the end of every session, participants were asked to undertake a homework assignment. This consisted of working with a carer to complete a ‘hassle log’, in which they described, analysed and evaluated a situation in which they had been angered that week. Around a third of each session, after the first, was devoted to discussion of one or two participants’ experiences, focusing primarily on ways in which the situation might have been handled differently to produce a better outcome, as contributed by facilitators and participants. | Breaks included. Role play to consolidate learning. Revision of materials covered in previous weeks (repetition). Inclusion of carers to help facilitate intervention and support homework tasks. | N/A | Improvements in anger ratings in EG which were maintained and further improved at 3 month follow up. | | | Further improvements at 3 month follow up | 4 | N/A | |
| 100. Barnett (2024) | Dissertation Qualitative - interpretive phenomenological analysis. | N=11 Age: 18-40 years Intellectual disability: Mild | Interviews were conducted to gain information about the perspectives of individuals with mild intellectual disability about mental health counselling  Interviews were conducted with people with mild intellectual disability. Interviews were concerned with their perspectives of mental health counselling. Questions were concerned with: - Expectations of counselling. - Personal and environmental contributions to change. - Therapeutic relationship. - Counselling process. - Outcomes from counselling. | Inclusion of family and other caregivers  - Reported to contribute to meaningful changes in participant lives outside of therapy.  Positive and negative experiences reported with counselling activities  - One participant said they did not offer support, and merely left toys to play with - Goal setting was seen as important  - Game-playing as well as talking were reported as valuable activities. - Activities described as 'helpful and inspiring' | N/A | N/A | | | N/A | N/A | N/A | |
| 101. Lake and MacHale (2022) | Journal article. Pilot study. Mixed-methods. | N=7 Age: 18-22 years Intellectual disability: Mild-moderate Stress  Country: Ireland | 2.5 hour mindfulness intervention (Based on Mindfulness-Based Stress Reduction practises) over 8 consecutive weeks. Sessions split in 2 parts to facilitate a coffee break. Part 1: - Entailed psychoeducation about stress and mental health with particular emphasis on anxiety, depression and anger. Part 2: - Comprised of mindfulness theory and various mindfulness exercises for their ease of use and understanding - Various types of mindfulness exercises were offered to expose participants to a range of different exercises that could be used independently.  Information about stress and mindfulness was introduced each week.  At the end of the 8 week block, each participant received a pack with an easy read summary of the MBSR group content. Pack contained: - a stress ball - a mood journal - a glitter jar - a certificate of achievement  - easy read information on mental health issues  - written instructions for all mindfulness exercises conducted during the group  - mindfulness imagery CD Group size: 7 | Easy read materials. Simplified language. Inclusion of supporters (supervisor from training centre) who attended weekly. | N/A | Significant improvement in well-being scores.  Improvement in self-esteem scores (not significant). | | | N/A | 5 | N/A | |
| 102. Yildiran and Holt (2015) | Journal article.  Thematic analysis. Qualitative. | N=6 Age: 21-46 years (mean=44 years) Intellectual disability: Mild-moderate paranoid personality disorder, recurrent depressive disorder, anxiety disorder and epilepsy Country: UK | Participants had to have attended between 2-23 sessions of mindfulness (mean number of attended sessions was 10).  One exercise was an adaptation of the raisin exercise (Kabat-Zinn 2012).  The group was facilitated by trainee and assistant clinical psychologists. It had been running for 1.5 years at the time the interviews took place for this study. | Multi sensory methods - using fruit as focal point - Participants were asked to focus on different sensory features of the fruit, the colour, smell, taste, texture, shape, sounds - used for p's to orient themselves in the present moment. Mindfulness-related exercises - Muscle tension and relaxation with a mindfulness element including a body scan where p's were prompted to notice changes in their bodies and locate where the warm feelings were, Olfactory experiences - incense sticks and candles used to focus on scents of fruits and flowers. | More sessions to be offered. Different or additional explanations and/or/techniques to be introduced. | Reported reductions in stress and emotional reactivity. | | | N/A | 5 | N/A | |
| 103. Ashworth et al. (2017) | Journal article. Case-study. Qualitative. | N=1 Age: Unknown Intellectual disability: Mild (IQ=67/69) Emotion regulation | Adapted DBT program developed to increase adaptive emotion management skills. Program includes: -Mindfulness -Managing feelings -Coping in crisis -People skills   Program ran every Monday for 2 hours with 15 minute breaks - facilitated by clinical psychologist and forensic psychologist in training. Program ran for 47 weeks. Participant attended 3 modules in group format and final module (9 sessions) individually with forensic psychologist in training.  - Attendance was at 91.5% overall. Group size: 3-9 (Average 5 members attending at one time) | Structured approach - consistency and familiarity with content and structure. Breaks included (15 minutes). Role-play to consolidate learning. | N/A | Some positive effect upon development and adaptive emotion management skills observed. - improved regulation of attention, awareness of experience and nonjudgmental attitude resulting in increased overall mindfulness index. However, improvement not reported by staff.  - Findings suggest a reduction in likelihood for participant to utilise maladaptive coping techniques. | | | N/A | N/A | 7 | |
| 104. Evans and Allez (2018) | Journal article. Case-study. Quantitative. | N=1 Age= 42 years Intellectual disability: Mild Low self-esteem | 15 sessions of CBT intervention included: -Psychoeducation -Mindfulness and distress tolerance strategies - Cultivating positive self-esteem - Activity scheduling - Working with the bottom line - It was hypothesised that the participant's 'bottom line' was that she is different because of her intellectual disability and mental health difficulties, as well as feelings of not being good enough. Efforts were made to understand where this belief came from and work with it. | Inclusion of prompts - prompt sheet of distress tolerance techniques was created to put on participant's bedroom wall. - This was done to minimise likelihood that participant would forgetting tolerance techniques. Inclusion of carer (staff member) to support participant outside of sessions.  - Carer would remind participant to complete homework and prompt participant to practice mindfulness and think about their positive qualities on a daily basis. Participant struggled with cognitive aspects of intervention, for example, generating alternatives to self-critical thoughts. - Alternative ways of doing this were implemented, such as self-critical statements being matched to kinder statements.  Visual aids included alongside verbal discussions to facilitate engagement and understanding. | More consistent support from staff (carers) during therapy. | Observed reduction in feelings of anger, and increase in self-esteem.  - Measured using adapted Rosenberg self-esteem scale. | | | N/A | 4 | N/A | |
| 105. Cooney et al. (2017) | Journal article. 2x3 (group x time) RCT: EG: cCBT group CG: TAU Pre-post data collected as well as 3-month follow up. Quantitative. | N=52 EG: N=26 CG: N=26 Age: 18+ years  Intellectual disability: Mild-moderate  Anxiety, depression, comorbid anxiety and depression, or recurring anxiety and depression  Country: Ireland | Computerised cognitive-behavioural therapy (cCBT). An adapted version of the Pesky gNATS CBT computer game and mobile application called "Pesky Gnats: The Feel God Island". The game teaches adults with intellectual disabilities and clinically significant anxiety or depression how to manage their mood via employment of general cognitive model.  -Simplifies core CBT concepts into a meaningful social story to Make it easier for participants with Intellectual disabilities to understand and engage with. - Played by an adult alongside a clinical psychologist   Participants took part in seven weekly sessions, each session lasting approx 1 hour.  During the game, players come across six different characters who teach seven core CBT skills across seven game levels. - Players complete a mindfulness or relaxation exercise presented as a video with audio at the end of each session. - Players complete between-therapy skills practice using the Pesky Gnats: Feel Good Island workbook. -Workbook is divided into seven chapters reflecting each level of the game  TAU: Received pharmacological treatment for anxiety and mood disorders. Ongoing psychological input provided by clinical psychologist included bereavement counselling for one participant, weekly individual CBT for anger management for one participant, weekly brief focused CBT for one participant. One participant in EG received counselling from a clinical psychologist during the study. | More psychoeducation using audio-visual methods. Workbook containing visual reminders of content of each therapy session.  - This is designed to support people to implement CBT, mindfulness and relaxation skills in their daily life at home, vocational setting or community. Each participant was supported by a clinical psychologist. Mindfulness and relaxation training at the end of each session. | N/A | Clinically significant decrease in anxiety symptoms in EG compared to TAU - Medium effect size post-intervention (d=0.67) -Large effect size at follow up (d=1.10) No significant reduction in depression symptoms. | | | 3-month follow up - Further reduction in anxiety symptoms with large effect size (d=1.10) | 4 | N/A | |
| 106. Mevissen et al. (2011) | Journal article.  Case series.  Qualitative. | N=4 Intellectual disability: Mild-moderate (IQ61-71) Age: 7-53 years  PTSD-like symptoms  Country: Netherlands | Weekly sessions of EMDR lasting 60 min each.  EMDR procedure adapted to level of clients' cognitive and emotional functioning. | Inclusion of supporters - professional caregiver to provide a sense of safety, to overcome communication disabilities, to facilitate an integration of the therapeutic process and daily life, and/or to function as a co-therapist. Visual cues used instead of abstract language (e.g. facial images to represent feelings, drawing to represent target memory if person is not able to mentally visualise it).  Physical gestures to aid communication (e.g. spreading arms apart or bringing them closer together to indicate amount of disturbance related to the target image). Where person is unable to perform eye movements, other forms of bilateral stimulation, primarily bilateral sound and tactile stimulation. From a developmental level of ±3 years downward the Story Telling Method is employed, with parents or caregivers narrating the story. The story will have a positive start, recognizable by the client. The next part of the story concentrates on the traumatic events, including distressing details the person might have seen, heard, felt, thought or smelled. The way in which the client had responded to the symptoms that emerged after the event was included. Finally, a recognisable positive ending is formulated as well as positive client self-beliefs. During the entire story, bilateral stimulation is applied. The story is repeated until the disturbance related to the target image has disappeared. An enjoyable game played between story narrations  - relaxes clients and emphasises safety and empowerment in present time, in contrast to temporary disturbance from the traumatic past. Beginning of each session includes a positive self-belief gleaned from some recent positive experiences may be installed. | N/A | Decrease in PTSD symptoms. | | | Results maintained at 3 months to 2.5 year follow-up. Depressive symptoms and physical complaints subsided and developmental growth increased, including social and adaptive skills. | N/A | 8 | |
| 107. Stenfert Kroese et al. (2016) | Journal article. Pilot study.  Mixed-methods. | Three groups: Group 1: N=3 Group 2: N=5 Group 3: N=4 All: Age: 21-46 years (mean=33 years) Intellectual disability: Mild Country: UK Trauma | Trauma-focussed cognitive behavoural therapy (CBT) - Stage 1 intervention for complex trauma aimed at promoting goals of personal safety, genuine self-care and capacity for healthy emotion-regulation  12 week programme (supplemented by additional sessions for consolidation) that includes: agreement on group rules, focussing on confidentiality; development of an individual formulation; staying safe and stabilisation; neuropsychology of trauma (how our bodies remember); feelings and thoughts (CBT model); understanding and managing emotions; improving relationships with others; depression and self-esteem; dissociation and different parts of ourselves; coping with triggers, memories, flashbacks and nightmares; self-harm and self-care; speaking up and moving on. | Sessions included role-play and mindfulness or relaxation exercises. Inclusion of carers, some of whom would attend group sessions. Art materials Exercise Flipcharts Role play | More Visual methods - DVDs Less information presented in short period of time. More time - longer sessions over longer periods of time. More information about how to cope with PTSD nightmares. Avoid assessments that may offend or give the impression that participants are judged. | Potential for intervention to decrease PTSD symptoms, however larger sample size required. | | | N/A | 5 | N/A | |
| 108. Hronis et al. (2024) | Journal article.  Case series. Quantitative. | N=11 Age: 8-17 years Intellectual disability: Mild-moderate-borderline Anxiety Country: Australia | 10 face-to-face sessions and an online program that breaks elements of CBT into their simplest form and provides participants with the opportunity to practice CBT skills through a range of exercises.  - Adaptations for potential deficits in attention, memory and learning, working memory, reading, and executive functioning.  There are three modules which children work through: one module which focuses on cognitions and cognitive challenging, a second module which focuses on behavioural changes and exposure, and a third module which teaches relaxation techniques. | Simplified elements of CBT - specific focus on breaking down cognitive components. Children begin by identifying and distinguishing between thoughts, feelings and behaviours, and practice this using case vignettes involving other children. They then move onto identifying and distinguishing between helpful and unhelpful thoughts, followed by learning to challenge their thinking. This process is facilitated by the online program which was designed as a way to make therapy more fun and engaging for the children, as well as a way to provide them with the opportunity to practice skills in a structured manner outside of the therapy sessions.  - Multi-modal approach (online and face-to-face) found to be useful with people with intellectual disabilities, especially with engagement, homework practice, and teaching skills and techniques. - Website has engaging visuals and illustrations, along with videos to help children understand concepts and practice relaxation techniques. - Short, simple sentences, often containing a single concept, with a text-to-speech function on the website for all written text in order to accommodate for deficits in learning and reading. - When completing tasks on the website, immediate feedback is provided to the child as to whether their response was correct or incorrect. - It is recommended that these tasks be completed with a therapist, or parents when completing homework, in order to discuss any mistakes.  -The vignettes on the website allows for repeated practice with different scenarios to facilitate the consolidation of each step in the cognitive challenging module. Parents are encour- aged to be actively involved in the process and to assist with homework where possible. Therapists are also able to run shorter therapy sessions than would be typical, or to include breaks in therapy to accommodate for possible deficits in attention. The program encourages that therapist implement adaptations to the therapy with flexibility, identifying what adaptations may be best suited to the individual child. | N/A | Significant reductions in at least one measure of anxiety or anxiety life interference.  - 6 children had significant reductions in anxiety on self-reported measure of anxiety. - 6 children had significant reductions in anxiety on the parent measure of anxiety. - 3 children had significant reductions in anxiety life interference. | | | N/A | 5 | N/A | |
| 109. Feniger-Schaal (2016) | Journal article. Case study. Qualitative. | N=1 Age: Unknown, however indications of being over 22 years old Intellectual disability: Moderate Schizophrenia, OCD | Weekly sessions of drama therapy lasting 50 mins each. 3 stages to therapy: First stage - seek common language - Objects, pictures, puppets and other projective means of expression. - Purpose to establish a relationship with client Second stage - Using images to express emotions. - Clinician took images that participant showed and made a story out of them that integrated the main ideas that stemmed from the images.  - Story mirrored feelings that participant had expressed in the images. This made participant feel seen.  Third stage - Story-making - Clinician offered participant an opportunity to create a story on their own.  - Scaffolding for the story was provided through the use of simple questions to guide participant.  - Over a period of 8 months, a story was created, and the story was read to participant at the beginning of every session.  - Participant was reported to be highly engaged with the process. Country: Israel | Visual methods - pictures to express emotion  - Pictures used to create stories - this narrative presented participant with an external representation of their inner world - enabled themes of aggression, victimisation and loneliness to be expressed and processed.  - Relationship and technique allowed participant to address these themes as the narrator and not only as the victim, moving from a passive position to a more active one. | N/A | After 3 years of therapy, participant presented significant relief in his psychiatric symptoms and improvement in general functioning. OCD behaviour diminished and was almost unseen Participant seemed stronger, livelier and less fragile. Participant communicated more openly with his surroundings, including parents, social worker, and made new friends. | | | N/A | N/A | 6 | |
| 110. Jahoda et al. (2017) | Journal article. RCT EG: Behavioural activation intervention (BeatIt) CG: Guided self-help (StepUP) Quantitative. | N= 161 Age: 18+ years  Intellectual disability: Mild-moderate (Mean IQ= 55.44 (SD=8.02) Depression Country: UK EG: N=84 CG: N= 77 | EG: 12 sessions manualised approach (BeatIt intervention) Focus of intervention: - Increasing activity - Scheduling activity - Addressing barriers to achieving engagement in activity First stage involved obtaining insight into participants' pattern of life and sharing formulation of their difficulties before working to increase participants' engagement and overcome emotional and organisational barriers to change. - Therapy ended with a plan to maintain or continue progress  CG: 8 session guided self-help intervention (StepUp). Structured, manualised approach with presence of a supporter - More psychoeducational and less person-centred  After building rapport in first session, a series of four booklets provided focus for next five sessions -Booklets concerned depression and factors linked to low mood, sleep, physical activity, and problem solving.  Therapy finished with review of all booklets and then a final session making a plan for their continued use. | Inclusion of supporters  - Supporters had to be available at least 2 hours per week.  - People with intellectual disabilities required supporters, when taking part in BeatIt, to engage in activity and negotiate change. - People taking part in StepUp required help with understanding booklets and using them in their everyday lives.  Supporters also helped ensure that people had help maintaining therapeutic gains once the interventions had finished. | N/A | No evidence of effectiveness of BeatIt compared with StepUp. Both groups showed significant reductions in depressive symptoms. | | | 4 months - within-group observed improvements  12 months - Improvements maintained | 5 | N/A | |
| 111. Haddock et al. (2004) | Journal article.  Multiple-case single-case experimental design.  Quantitative. | N=5 Age: 21-53 years  Intellectual disability: Mild Schizophrenia Country: UK | 20 weekly sessions (5 engagement and 15 intervention) over 6 months lasting between 30 and 60 minutes each. Delivered one to one (except where family members were involved) Intervention was based on the Manchester model of CBT for psychosis. Initial sessions focused on the assessment of core symptoms, formulation of key areas for intervention and the application of cognitive behavioural strategies to deal with hallucinations, delusions, negative symptoms and associated difficulties of anxiety, depression and social functioning. Specific strategies included: - Psychoeducation. - Belief modification. - Reality testing.  - Strategies for managing auditory hallucinations. - Activity scheduling.  - Coping strategy enhancement. | Specific family intervention strategies were used (modified from Barrowclough and Tarrier, 1992) where a family member played an important role in a person's care.  Visual methods - pictures to illustrate concepts and help discuss emotions. - Also helped combat issues with reading and writing. | N/A | Little change in PANSS positive scores between baseline and end of treatment. | | | N/A | 5 | N/A | |
| 112. Oathamshaw et al. (2013) | Book chapter  Descriptive | N=1 Age: late 50s Intellectual disabiltiy: Mild Psychosis | CBT included: - Seeking a shared experience of internal speech and thought - Seeking to normalise internal experiences - Normalisation of negative content  - Attentional biases and ego-dystonic thoughts - Mindfulness exercises - Reframing negative attentional biases - Reflections on memory activation and frequency of distressing thoughts | Simplified language Visual representations of abstract concepts Tactile stimuli where applicable  Involvement of family members and caregivers | N/A | N/A | | | N/A | N/A | N/A | |

| **First Author (Year)** | **Study type** | **Participants** | **Intervention** | **Adaptation** | **Future adaptation recommendations** | **Outcome** | **Follow up** | **Quality of paper (MMAT)** | **Quality of paper (JBi case series)** |
| --- | --- | --- | --- | --- | --- | --- | --- | --- | --- |
| 113. Jahoda et al. (2024) | Journal article  Behavioural activation exemplar (BeatIt) | Intellectual disability: Severe/profound  Depression | 12 sessions manualised behavioural activation intervention.  Therapy involved:  - Goal setting.  - Activity scheduling.  - Addressing individual, inter-personal and systemic barriers to change. | Structured approach,  Accessible language.  Use of visual materials.  Positive reinforcement.  Inclusion of supporters (family members and paid carers) to help assess the needs of people with intellectual disabilities to inform adaptations required during therapy.  Easy-read materials.  Incorporation of activities between sessions, developed with supporters to tailor to the needs and desires of individuals with intellectual disabilities. Supporters will be involved in completing therapeutic tasks that are unable to be completed by clients with intellectual disabilities alone.  Incorporation of mood diaries.  Filming people with intellectual disabilities’ involvement in activities and scheduled homework tasks (ensures their voices are heard).  - Showing excerpts from these films can be used to illustrate ways of supporting individuals to engage in activities, as well as their impact on mood. | N/A | N/A | N/A | N/A | N/A |
| 114. Pineda et al. (2023) | Journal article.  Qualitative.  Descriptive. | 12 adults  Williams syndrome  Anxiety | Adapted CBT:  12 group sessions delivered twice per week. | - Concrete techniques with clear and simple instructions (simplified language).  - Inclusion of carers as a therapy partner who were chosen by participants and included parents and/or group home staff. Therapy partners helped facilitate a participant’s attendance and engage in sessions, timelines, completion of homework, and application of skills outside of group sessions.  - Incorporating repetition.  - Slowing pace of instruction.  - Using specific examples and adaptations for WS.  - Using a virtual group format (online method).  - Adapted child-based CBT to tailor to adults with WS/ID.  - Included visual methods (e.g. cartoon thought bubbles).  - Included images/metaphors relevant to adult’s everyday life adapted from child-based CBT manuals (e.g. describing times when people would feel anxious at work rather than at school). | Future research should focus on identifying which aspects of CBT are most effective for adults with intellectual disabilities including: 1) whether formal inclusion of exposures improves treatment outcomes, 2) identification of treatment modifiers such as cognitive and/or language ability, 3) investigation of underlying treatment mechanisms, 4) how to most effectively incorporate caregiver involvement, and 5) whether treatment gains are maintained over time. | N/A | N/A | N/A | N/A |
| 115. Montanaro et al. (2024) | Journal article.  Pilot study.  Quantitative. | N=10  Mean age: 12.09 (SD=2.6)  Intellectual disability: Mild, moderate, severe  Depression, Anxiety. | CoM-II conducted by combining cognitive behavioural training and neuropsychological training.  - Eleven sessions (including one online session carried out by caregivers)  - Ten in-person sessions lasted 5 hours with a 30 min lunch break. These meetings focussed on improving socio-pragmatic and communicative skills, as well as cognitive abilities, emotional management and adaptive functioning.  - Video calls lasted approximately 60-90 minutes, and participants were paired based on their cognitive strengths and weaknesses. | Each session began with group psychological support – it was important for participants to share the most salient emotional events of the previous month with the therapist.  Each in-person meeting included psychoeducation. – Therapist carried out didactic lessons by utilising written and video materials specifically designed for participants.  Inclusion of carers to facilitate therapeutic process.  Educators trained by therapists: assisted with role-playing demonstrations and carried out video-calls between sessions.  Homework assignments consisted of socialisation activities, cognitive behavioural exercises with a particular focus on emotional management, neuropsychological trainings combining material extracted from specific books, and exercises created by therapists. | N/A | Decrease in depressive and anxiety symptoms.  - Increase in socio-pragmatic and communication skills from pre-to-post-test.  - Improvement in adaptive behaviour of participants and in family quality of life. | Effects maintained at follow-up. | 5 | N/A |
| 116. Alilou and Maleki (2024) | Journal article  Behavioural activation therapy.  Case study.  Quantitative. | N=2  Age: 21-22 years  Intellectual disability: Borderline  Depression, Anxiety | 12 sessions of behavioural activation therapy. Sessions held in person for the first person, and online (WhatsApp video calls) sessions for the second person. Duration of sessions varied from 1hr to 1hr 30 min. Sessions were held twice a week at the beginning, but once a week as final sessions approached. | Simplified materials (e.g. simplified images and added images to fit the needs of people with borderline intellectual disability). | N/A | Participant A showed more progress in depression than subject B. Subject B experienced more improvement in anxiety than subject A. | One month follow up effects maintained. | 5 | N/A |
| 117. O'Riordan et al. (2024) | Journal article. .  Qualitative.  Descriptive. | N=8  Intellectual disability: unspecified  Grief | Eight interviews conducted with four psychiatrists, two psychologists, an advanced nurse practitioner and a social worker. | Pre clinical sessions to build rapport with client, as well as plan and explain the therapeutic process with clients. – Information included within this session will be repeated each session.  Accessible handouts provided to give people with intellectual disabilities an overview of the therapy.  Consideration of the structure of therapy for potential fatigue, and the time needed to address complex concepts with individuals. – CGT was therefore split into two sessions (Sessions 1A and 1B).  Session 1A: includes continuing the development of rapport, discussing the relationship with the deceased and story of the death, reviewing the responses to the CGQ-ID, and completing an adapted version of the Grief Supports Inventory.  Sesion 1B: Introduces the idea and process of grief monitoring and interval plans, which allow the individual to identify things they will do between sessions that reflect things they enjoy.  The ‘grief partner’- a more constant support person than proposed by the original model, and could be a key worker or staff member.  Simplified concepts and materials to ensure accessibility of sessions for people with intellectual disabilities. –Simplified language and concepts as well as use of visual materials. Emphasis placed on ‘imaginal revisiting’. This involves supporting the individual to remember something or to physically engage with something (e.g. place of activity) that is linked to the deceased person. Imaginal revisiting invites the bereaved individual to tell a story of the death, which is recorded for later review and reflection. | Further development of accessible materials – simplify language and concepts to support comprehension. | N/A | N/A | 5 | N/A |
| 118. O’Farrell et al (2024) | Journal article  Within subjects pre-post design  Quantitative | N=10  Age: 18-45 years  Intellectual disability: unspecified  Trauma, PTSD | Children’s accelerated trauma technique (CATT) – a trauma focused CBT-based approach.  12 steps were followed, one additional step ‘six-part story making exercise’ was added.  12-18 weekly sessions were delivered. | ‘Six-part story making exercise’ added before starting standard CATT steps to assess whether people understood and engaged with narrative ‘replay’ component of therapy.  Therapy was delivered face-to-face in a variety of settings (e.g. art rooms, hired community spaces).  Flexible approach to therapy – number of sessions varied from 12 to 18 to accommodate individual needs.  Therapy used art (visual methods) and storytelling to improve accessibility for people with intellectual disabilities. | N/A | Significant reduction in trauma symptoms. | N/A | 3 | N/A |
| 119. Langdon et al (2024)  -Published in Health Technology Assessment (HTA) and Journal of Applied Research in Intellectual Disabilities (JARID). | Journal article  Single-arm non-randomised pre-and-post test feasibility study.  Mixed-methods. | N=28  Age: M= 26.79 years (SD= 8.39)  Intellectual disability: moderate-severe  Anxiety | BEAMS-ID (Behavioural interventions for Anxiety in adults with Autism and Moderate to Severe Intellectual Disabilities).  - A manualised behavioural therapy adapted specifically for adults with autism and moderate to severe intellectual disabilities who experience anxiety.  Participants took part in 12 sessions, each lasting 60-90 minutes delivered over several weeks within NHS community settings.  Grounded in behavioural principles and includes components of relaxation training, fear hierarchy development, exposure therapy, and behavioural experiments. | Carers or family members involved in the delivery of the intervention.  Use of methods less reliant upon verbal communication – graded exposure techniques coupled with relaxation and reinforcement.  Visual methods –visual schedules.  Simplified language - easy-read materials using clear, simple and direct language. Complex psychological terms avoided.  Talking mats used during interviews to support communication and understanding.  Intervention flexibility – timing, frequency and number of sessions were adapted to suit individual needs. – therapists were encouraged to be responsive to attention span, engagement and cognitive capacity.  Repetition of key concepts taught during therapy, and homework included practising relaxation, repeating a step from fear ladder, and progress tracking. | Carers suggested that intervention could be delivered over a longer period. One carer suggested shorter sessions | N/A | N/A | 5 | N/A |
| 120. Hoogstad & Mevissen (2024) | Journal article  Multiple-baseline single-case experimental design.  Quantitative. | N=3  Age: 30, 31 and 35 years.  Intellectual disability: Severe  PTSD | EMDR storytelling method – adaptation of standard EMDR protocol for children ages under 4 years, matching cognitive developmental age of people with severe intellectual disability.  - 60 minutes of EMDR delivered once per week. | Inclusion of carers (paid and relative).  Traumatic memory activated by offering a story about event, while at the same time, a distracting task (e.g. tactile or auditory).  Additional resources (e.g. pictures, touch and objects). | N/A | Reductions in symptoms of PTSD | Effects maintained at 3-month follow up. | 3 | N/A |
| 121. Gray et al (2024) | Journal article  Single-group non-randomised pre- and-post test feasibility study.  Mixed-methods. | N=14  Age: 5-15 years  Intellectual disability: Moderate-severe.  Phobia | Parent-mediated exposure-based program designed for children with intellectual disabilities.  Developed using co-production with parents, therapists and researchers. Two parent skills training workshops (group delivery), followed by  eight weekly individual sessions conducted via phone or video call – therapists guide parents in implementing exposure tasks.  Sessions lasted 30 mins. | Augmentative and alternative communication strategies – use of visual aids, manual signs and gestures (non-verbal), and concrete language.  Visual schedules.  Inclusion of carers (parents).  Breaks included during therapy.  Relaxation techniques integrated during therapy.  Blowing bubbles, fidget things and sensory items added as alternatives to standard relaxation exercises.  ‘Easy on the I’ images used.  Delivered via telephone/video call. As well as group sessions.  Use of prompts.  Therapists advised parents individually.  Shorter sessions. | More flexibility in the structure of therapy.  More sessions. | N/A | N/A | 5 | N/A |

**Table S2**

*An initial framework for adapting psychological interventions that was developed using NICE (2016) guidelines.*

| **Targeted Mental Health Problem** | **Contextual Alterations** | **Language** | **Support** | **Mode of Delivery** | **Intervention Flexibility** |
| --- | --- | --- | --- | --- | --- |
|  | Individual Preferences  Sensory Preferences | Simplification | Family  Caregiver | In Person  Online  Telephone | Session Time Increase  Session Time Decrease  Session Breaks  Extra Introductory Sessions |

**Table S3**

*Final framework:*

| **Category** | **Description** |
| --- | --- |
| **Multi-sensory methods** | Incorporating therapeutic techniques that engage a variety of senses (e.g. audio-visual methods). |
|  |  |
|  |  |
|  |  |
| **Activities** | Incorporating activities during therapy (e.g. physical activities, games) |
|  |  |
|  |  |
|  |  |
| **Communication** | Alter communication by simplifying language and concepts as well as incorporating non-verbal components (e.g. exaggerating facial expressions). Clinicians may use a directive approach, using prompts and positive reinforcement to aid with understanding and retention of concepts. |
|  |  |
|  |  |
|  |  |
|  |  |
|  |  |
|  |  |
| **Delivery medium** | Be mindful of the ways in which therapy is delivered, whether face-to-face, online, multi-modal, group setting or individual setting. Clinicians may also incorporate technology during therapy (e.g. robot, heart monitor, etc), and may also conduct therapy in different environments, be it a clinic, outdoors, or a client’s house. |
|  |  |
|  |  |
|  |  |
|  |  |
|  |  |
|  |  |
|  |  |
|  |  |
| **Additional support** | Inclusion of supporters during therapy. Supporters could aid with therapy both during and outside of sessions. |
|  |  |
|  |  |
| **Structure** | Clinicians should be mindful of the ways in which psychotherapies are structured. These will need to be tailored to the individual needs of clients and could include shorter sessions, longer sessions, more sessions, less sessions, increased frequency, decreased frequency and content reduction. Breaks may also be included depending how long clients can stay engaged for. Some clients may benefit from a structured approach, whereas others may prefer a more flexible approach to treatment. Additional psychoeducation, frequent progress tracking, and more homework may aid in retention of learned concepts during therapy. Additional resources, such as easy-read materials, may also be incorporated during therapy. Group sizes may need to be reduced to focus more on the individual needs of clients, and clinicians may consider incorporating relaxation exercises to help moderate emotions. |
|  |  |
|  |  |
|  |  |
|  |  |
|  |  |
|  |  |
|  |  |
|  |  |
|  |  |
|  |  |
|  |  |
|  |  |
|  |  |
|  |  |
|  |  |
|  |  |
|  |  |
